# A Soft Matrix Microenvironment Promotes Laterally Spreading Tumors via Oxidative Phosphorylation‐Dependent Cell Adhesion

**DOI:** 10.1002/advs.202523872

**Published:** 2026-03-15

**Authors:** Jiamin Zhong, Jingyi Lu, Haopeng Li, Jun Zhong, Xiaobei Luo, Yiyi Hu, Xianfei Wang, Pengfei Wang, Yanning Zhang, Zhenjiang Wang, Qiuhua Lai, Zhenyu Chen, Wenting Mi, Wang Tin San‐to, Weize Li, Shuping Tan, Qihong Cheng, Ruijia Li, Yida Nie, Side Liu, Bing Huang, Zelong Han

**Affiliations:** ^1^ Guangdong Provincial Key Laboratory of Gastroenterology Department of Gastroenterology Nanfang Hospital Southern Medical University Guangzhou China; ^2^ Department of Gastroenterology and Department of VIP The Eighth Affiliated Hospital of Southern Medical University (The First People's Hospital of Shunde, Foshan) Foshan China; ^3^ Digestive Endoscopy Center, Department of Gastroenterology Affiliated Hospital of North Sichuan Medical College Nanchong China; ^4^ Department of Gastroenterology The Second Hospital & Clinical Medical School Lanzhou University Lanzhou China; ^5^ Department of Gastroenterology Zhuhai People's Hospital (Zhuhai Clinical Medical College of Jinan University) Zhuhai China

**Keywords:** adenomas, colorectal neoplasms, extracellular matrix, laterally spreading tumors, oxidative phosphorylation, tumor microenvironment

## Abstract

Laterally spreading tumors (LSTs) are large, flat, early‐stage precancerous colorectal lesions that are frequently overlooked during endoscopic examination and present distinct clinical challenges compared to conventional protruding adenomas (PAs). Despite similar histology, the distinct lateral growth of LSTs suggest underlying molecular differences that remain poorly understood. Here, we comprehensively profiled the molecular signatures, cellular phenotypes, and tumor microenvironments of LSTs, PAs, and adjacent normal tissues (NTs) using single‐cell RNA sequencing and spatial transcriptomics, clinical specimens and patient‐derived organoid models. LSTs exhibited a more malignant phenotype characterized by transcriptome‐inferred high copy number variation (CNV) scores, stronger genetic correlation with colorectal cancer, and downregulation of adhesion molecules. Transcriptomic analyses revealed that this downregulation is closely associated with cytoskeletal depolymerization and enhanced oxidative phosphorylation (OXPHOS). Notably, LSTs reside in a softer extracellular matrix than PAs; organoid modeling indicated this environment promotes OXPHOS and modulates adhesion via the ENTPD1–ADORA2B axis. Integrating these observations, we propose a mechanochemical model where a soft matrix is coupled with OXPHOS and cytoskeletal remodeling through ENTPD1–ADORA2B, coinciding with adhesion suppression. These findings provide integrative insights into potential regulatory dynamics underlying LST lateral growth and highlight the ENTPD1–ADORA2B axis for future mechanistic investigation.

## Graphical abstract

Laterally spreading tumors (LSTs) are precancerous colorectal lesions characterized by a flat morphology. This study reveals a mechanochemical pathway through which a soft matrix microenvironment diminishes spatial constraints in intestinal adenomas. This process promotes deficiencies in tight junction proteins, mediated by the mechanoreceptor ADORA2B and associated with enhanced oxidative phosphorylation and cytoskeletal dissolution, ultimately facilitating the lateral spread of adenomas.

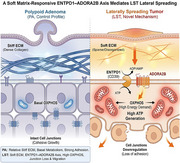

**Abbreviations**
ASC: adenoma‐specific cells
COLLAGEN I: collagen type I
CRC: colorectal cancer
CytoD: Cytochalasin D
DEGs: differentially expressed genes
ECM: extracellular matrix
Epi‐D: deep layer of epithelium
Epi‐M: middle layer of epithelium
Epi‐S: superficial layer of epithelium
FC: fold change
H&E: hematoxylin and eosin
IHC: immunohistochemical
Immune: immune regions
JNET: Japan NBI Expert Team
LST: Laterally spreading tumor
NICE: NBI International Colorectal Endoscopic
NMF: non‐negative matrix factorization
NT: normal mucosa
OXPHOS: oxidative phosphorylation
PA: protruded adenoma
PPV: type V of pit pattern classification
SEM: scanning electron microscopy
ST: spatial transcriptomic
Stromal: stromal regions
TEM: Transmission electron microscopy
TJs: tight junctions
MMP: matrix metalloproteinase
SR: strain ratio
α‐SMA: α‐smooth muscle actin
WGCNA: weighted gene correlation network analysis.

## 1 Introduction

Laterally spreading tumors (LSTs) represent a distinct type of superficial precancerous lesion in the large intestine, characterized by lateral expansion exceeding 10 mm along the mucosal surface or circumferentially along the bowel wall [1, 2]. Compared with conventional protruding adenomas (PAs), LSTs are more challenging to detect during early endoscopic screening due to their flat morphology and subtle mucosal features [3, 4]. Notably, LSTs exhibit a higher malignant potential with an increased risk of submucosal invasion and local recurrence after endoscopic resection [5, 6]. Longitudinal studies have reported recurrence rates as high as 29% within 6 years [7]. Clinically, LSTs are stratified into Granular (LST‐G) and Non‐Granular (LST‐NG) subtypes based on surface morphology [8]. Notably, despite their superficial spreading pattern, the Non‐Granular type is associated with a significantly higher risk of submucosal invasion and malignancy compared to the Granular type, underscoring the heterogeneity of malignant potential within LSTs [5, 9, 10, 11]. Despite their clinical significance, the molecular and microenvironmental mechanisms underlying the characteristic lateral expansion of LSTs remain largely undefined. Recent studies have integrated single‐cell RNA sequencing (scRNA‐seq) data with large genome‐wide association studies (GWAS) to identify the cell‐of‐origin populations of colorectal cancer (CRC) within serrated polyps, adenomas [12] and CRC [13]. However, little is known about the potential relationship between hereditary factors associated with CRC risk and those involved in LST lesions, hampering advances in early detection and precise intervention.

The unique lateral growth pattern of LSTs implicates complex interactions between tumor biomechanics and the surrounding stromal environment. Tumor expansion and cellular deformability are tightly coupled to the biophysical properties of cancer cells and the extracellular matrix (ECM) [14, 15, 16]. In many solid tumors, progression is associated with increased ECM stiffness, often resulting from excessive collagen deposition and remodeling by cancer‐associated fibroblasts [17, 18]. However, paradoxically, highly metastatic tumor cells are often softer than their benign counterparts, with cancer stemness and altered differentiation states contributing to a more compliant tumor niche [19, 20], and dynamic rigidity changes can facilitate rapid cell migration on soft substrates [21]. In addition, adhesion reorganization plays a critical role in modulating cell‐matrix interactions and may provide a possible interpretation of mechanosensing activity involved in cell‐cell mechanical communication [22]. Whether these biomechanical alterations participate in the pathogenesis of LSTs remains unknown.

In this study, we investigated the cellular and molecular basis of LST progression with a particular focus on the stromal microenvironment. By integrating single‐cell transcriptomics, spatial transcriptomics, and functional analyses using patient‐derived organoids, we characterized the transcriptomic programs associated with LST‐specific growth. Our findings revealed that the downregulation of adhesion molecules is closely coupled with signatures of enhanced oxidative phosphorylation (OXPHOS)in LSTs. Furthermore, we observed that a softer extracellular matrix (ECM) coincides with this metabolic shift and the attenuation of adhesion programs, potentially reducing spatial constraints on tumor growth to favor lateral expansion. Integrating these empirical observations, we propose a mechanochemical model for LSTs. These insights provide a foundational framework for understanding the lateral expansion dynamics and offer potential avenues for early diagnosis and targeted therapy.

## 2 Results

### 2.1 A Multidimensional Analysis Reveals Signatures Associated With the Higher Malignant Potential of LSTs

**FIGURE 1:**
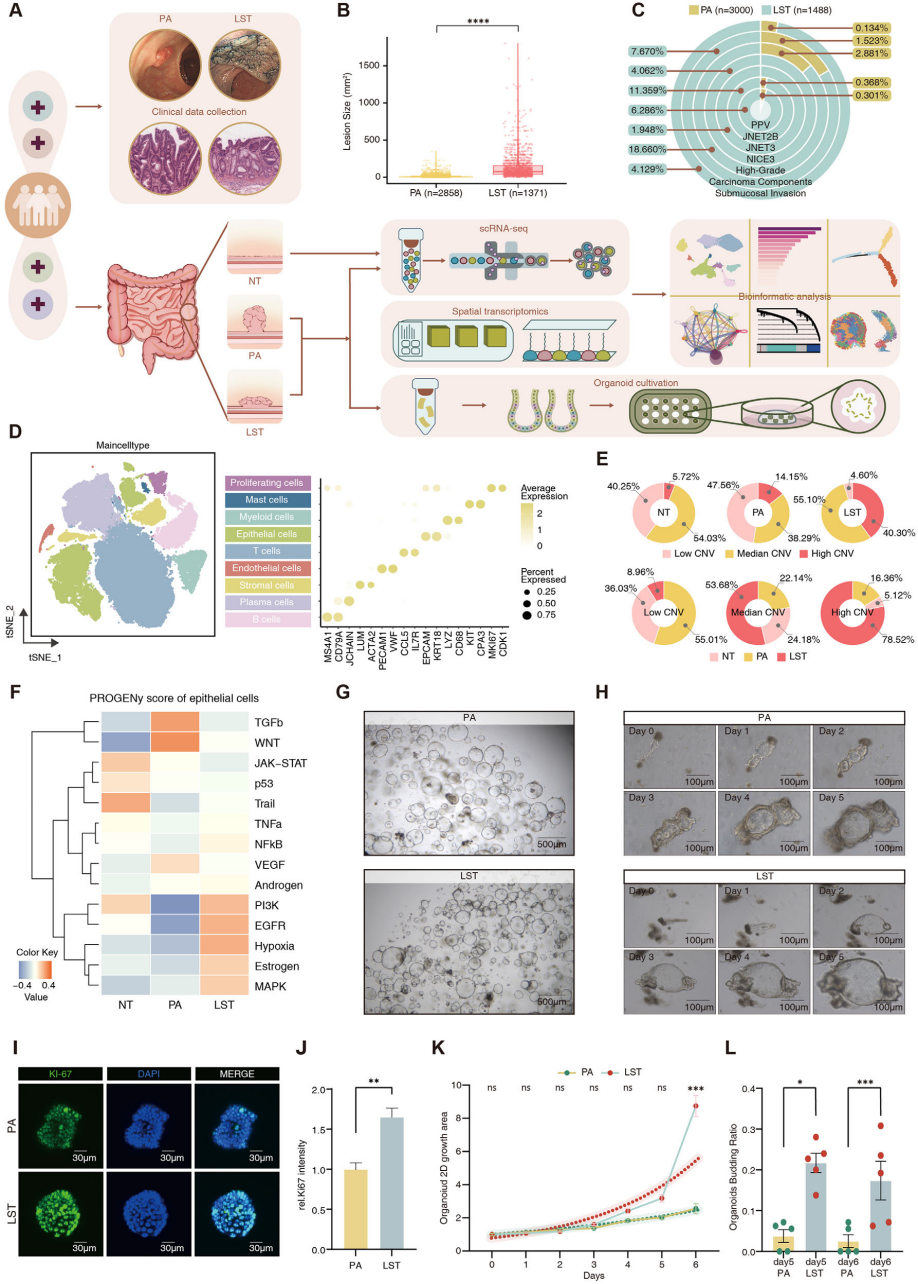
Elevated malignant potential in LSTs. (A) Study workflow outlining the experimental design. (B) Bar chart presenting the statistical analysis of lesion areas in PA (n=2858) and LST (n=1371) groups, with each dot representing data from individual patients. (C) Pie chart depicting the proportion of cases for clinical indicators in PAs (n=3000, yellow) and LSTs (n=1488, blue). (D) tSNE visualization displaying clusters by major cell types (left) and a bubble plot indicating marker gene expression levels across cell subtypes (right). (E) Pie charts depicting the distribution of NT, PA, and LST epithelial cells among CNV‐defined clusters (top), and the distribution of these CNV‐defined clusters within NT, PA, and LST samples (bottom) with explicit percentages. (F) Heatmap showing distinct pathway activities in epithelial cells across three groups as inferred by PROGENy. (G) Representative bright‐field images of organoids derived from PAs and LSTs at day 7 of culture, at 4× magnification. (H) Representative bright‐field images demonstrating growth of PA and LST organoids over five consecutive days, at 20× magnification (n=5 for each group). (I) Representative immunofluorescence images of organoids (n=3) stained for Ki‐67. (J) Bar plot displaying the relative intensity of immunofluorescence signals in (I). (K) Relative area of PA (green) and LST (red) organoids from day 0 to day 6, modeled with second‐order polynomial fitting (n=4 for each group). *p* values were calculated using the two‐way ANOVA, followed by Sidak's multiple comparisons test. (L) Budding ratio of PA (yellow) and LST (blue) organoids on day 5 and 6, with each dot representing the budding rate of an individual image (n=5 for each group). All *p* values were calculated using the Mann‐Whitney U test unless otherwise stated. *p < 0.05, **p < 0.01, ***p < 0.001, ****p < 0.0001.

To assess the malignant potential of LSTs, we analyzed the clinical and histopathological data from 4488 cases collected across four medical centers (Figure 1A; Figure S1A,B). On average, LSTs were approximately ten times larger in size than PAs (Figure 1B) and were more frequently classified as high‐risk endoscopic subtypes, including the Type V pit pattern (PPV), NBI International Colorectal Endoscopic (NICE) type III, and Japan NBI Expert Team (JNET) type 2 B or 3. Histological evaluation further confirmed a significantly higher incidence of high‐grade dysplasia, carcinoma components, and submucosal invasion in LSTs than in PAs (Figure 1C).

To explore whether transcriptomic signatures from independent cohorts align with this malignant phenotype, we analyzed public bulk transcriptomic datasets comprising PA and LST samples. Despite the inherent heterogeneity of these datasets, weighted gene co‐expression network analysis (WGCNA) identified a distinct LST‐associated module (MEbrown) (Figure S1C–E). This module was enriched for malignancy‐related transcriptional programs, including those implicated in gastric adenocarcinoma, invasive breast carcinoma, recurrent tumors, and esophageal adenocarcinoma (Figure S1F). In contrast, the PA‐associated module (MEturquoise) lacked such enrichment (Figure S1G). These observations provide supplementary molecular context that is consistent with the clinically aggressive nature of LSTs.

To obtain a comprehensive view of the cellular and molecular architecture, we integrated in‐house scRNA‐seq data with publicly available datasets, analyzing a total of 21 samples across three groups (7 NT, 7 PAs, and 7 LSTs; see Supplementary Table S1). This integrated cohort yielded 90 134 high‐quality transcriptomes.(Figure 1A; Figure S1H). Based on canonical marker genes, we annotated nine major cell populations: epithelial cells (*EPCAM*, *KRT18*), plasma cells (*JCHAIN*), B cells (*MS4A1*, *CD79A*), stromal cells (*LUM*, *ACTA2*), T cells (*CCL5*, *IL7R*), myeloid cells (*CD68*, *LYZ*), endothelial cells (*PECAM1*, *VWF*), Mast cells (*KIT*, *CPA3*), and proliferating cells (*MKI67*, *CDK1*) (Figure 1D; Figure S1I and Table S2A). These populations were consistently detected across all samples (Figure S1J), reflecting their in situ cellular composition. Transcriptome‐inferred copy number variation (CNV) scores were evaluated across all epithelial cells, which were subsequently stratified into three categories—low, medium, and high CNV levels. Notably, LST samples were enriched for epithelial populations with medium and high CNV levels, over half of which originated from LSTs (Figure 1E). Furthermore, tumor‐related pathway scoring using PROGENy revealed marked activation of multiple oncogenic signaling cascades, including PI3K, EGFR, hypoxia, and MAPK pathways, in LST epithelial cells, consistent with a more malignant phenotype (Figure 1F).

To recapitulate the growth behavior of LSTs in vitro and to explore the associated cellular phenotypes, we established patient‐derived organoids from LST and PA lesions (Figure 1G,H). These organoids preserved key histological features of their respective tumors, including epithelial origin and goblet cell differentiation, as confirmed by scanning electron microscopy (SEM) and periodic acid‐Schiff Alcian blue staining (Figure S2A,B). Notably, LST‐derived organoids exhibited significantly elevated Ki‐67 expression (Figure 1I), as well as markedly greater lateral area expansion and higher budding ratios compared with their PA‐derived counterparts (Figure 1K,L; Figure S2C). The increased budding ratio showed a strong correlation with overall tumor growth [23, 24].

Collectively, these results highlight transcriptomic and phenotypic features that align with the higher malignant potential of LSTs, as reflected by their larger size, aggressive pathological features, enriched malignancy‐associated transcriptional networks, and more invasive behavior in organoid models.

### 2.2 Deciphering the Transcriptomic Features and Evolutionary Landscape of Epithelial Cells

**FIGURE 2:**
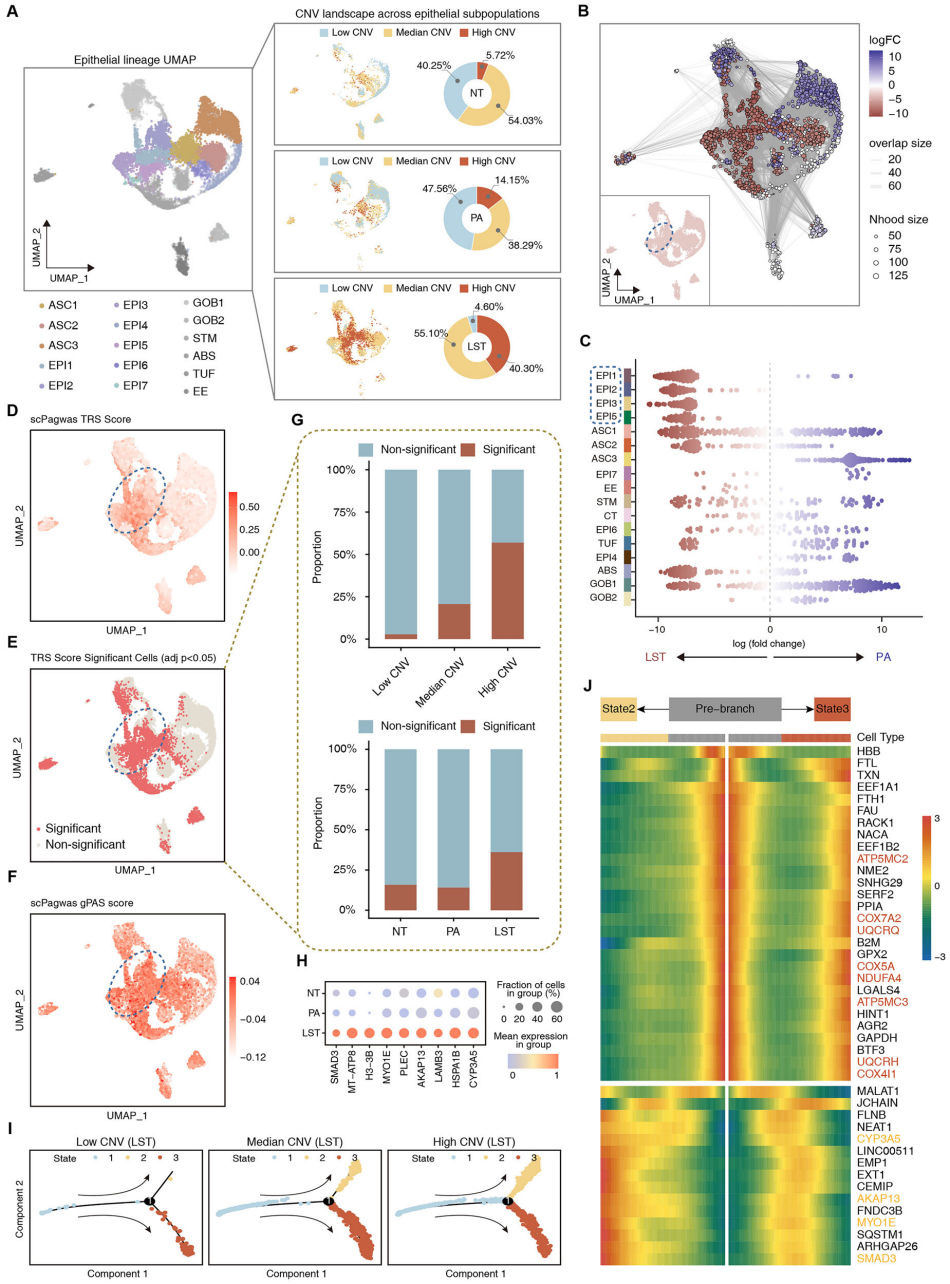
Unique molecular characteristics of the LST epithelium. (A) UMAP plot of epithelial cells colored by subtype (left). Right: Distribution of epithelial cells across NT, PA, and LST groups, stratified by CNV states and relative proportions. (B) A neighborhood graph was constructed using MiloR, where each node represents transcriptionally similar cells and edges indicate shared cells between neighborhoods. Node positions correspond to the mean UMAP coordinates of their constituent cells, and colors denote the predominant cell type within each neighborhood. The landscape of epithelial cells is shown in the lower‐left panel, with dashed circles highlighting subpopulations specifically enriched in LSTs. (C) Beeswarmplot showing the differential abundance across epithelial subtypes; EPI1, EPI2, EPI3, and EPI5 showing significant differences in abundance between PAs and LSTs. (D) UMAP visualization of the inheritance‐related enrichment score generated by scPAGWAS, representing the relative distribution of genetics‐associated signals across epithelial cells. (LST‐specific subpopulations are demarcated by the dashed circle) (E) UMAP visualization of cell‐wise statistical significance of the TRS score as defined by scPAGWAS, with genetically significant cells highlighted in red. (LST‐specific subpopulations are demarcated by the dashed circle) (F) UMAP plot showing the inheritance pathway regression effect score inferred by scPAGWAS for each epithelial cell. (LST‐specific subpopulations are demarcated by the dashed circle) (G) Bar plot showing the proportion of significant cells across CNV‐defined groups (top) and tissue types (bottom). (H) Dot plot illustrating the expression of top heritability‐correlated genes across the three groups. (I) Pseudotime trajectory of cells across different CNV‐defined groups, with colors indicating distinct cell states. (J) Heatmap depicting genes with significant branch‐dependent expression. Columns represent points along pseudotime, rows represent genes, and the pseudotime origin is positioned at the center of the heatmap. From the center, progression to the left corresponds to the cell state 2 trajectory, while progression to the right corresponds to the cell state 3 trajectory.

To gain deeper insights into the molecular features of LSTs, we first delineated 17 distinct epithelial subtypes [25], including adenoma‐specific cells (*AXIN2*, *RNF43*), goblet cells (*TFF3*, *MUC2*)), stem cells (*ASCL2, LGR5, OLFM4*), absorptive cells (*KRT20*, *GUCA2A*), tuft cells (*POU2F3*, *SOX9*), crypt top colonocytes (*MEIS1*, *OTOP2*), enteroendocrine cells (*NEUROD1*, *CHGA*) and several other clusters with distinct features (Figure 2A; Figure S2D and Table S2B). The distribution of these subtypes and their corresponding inferred CNV levels are shown.

Differential abundance analysis revealed tissue‐specific preferences of adenoma‐specific cells (ASCs): ASC1 and ASC2 were present across all three groups but predominated in LSTs, whereas ASC3 was mainly detected in PAs. Clusters EPI1, EPI2, EPI3, and EPI5 were notably enriched in LSTs (Figure 2B,C; Figure S2E) and corresponded to cells with higher CRC risk scores, as determined by scPagwas, a computational framework that integrates single‐cell transcriptomes with GWAS data to identify disease‐associated cell populations (Figure 2D–F). Proportion analysis further revealed that CRC risk–related positive cells were preferentially enriched in populations with higher inferred CNV scores (Figure 2G; Figure S2F), and top heritability‐associated genes were highly expressed in LST epithelial cells (Figure 2H; Figure S2G). Group‐specific pathway enrichment via scPagwas revealed that LST epithelial cells were characterized by glycan degradation and metabolic processes including OXPHOS. We hypothesize that the glycan degradation pathway could participate in ECM remodeling, reducing matrix stiffness and thereby facilitating cell motility (Figure S2H).

We further examined the functional implications of cells stratified by inferred CNV levels in PA and LST cells: LST cells with predicted medium and high CNV scores were associated with OXPHOS, diverse mitochondrial processes, and regulation of CDH1 (E‐cadherin, a key cell adhesion molecule) expression and function (Figure S2I,J). In contrast, cytoskeleton organization–related processes were more frequently enriched in PA cells with comparable inferred high CNV scores (Figure S2K), suggesting a relative attenuation of these programs in LST epithelial cells.

To trace the developmental roles of cells with varying inferred CNV profiles during LST progression, we performed pseudotime analysis, revealing a distinct differentiation trajectory: low‐inferred‐CNV cells predominantly occupied early states, whereas medium‐ and high‐inferred‐CNV cells were enriched at terminal states (Figure 2I; Figure S2L). BEAM analysis further identified differentially expressed genes (DEGs) between the two fates. Notably, pseudotime dynamics revealed a coordinated divergence between metabolic and oncogenic programs rather than a simple inverse correlation. Genes associated with CRC risk followed a ‘Low‐High‐Low’ pattern, peaking at the intermediate transition state (State 2). Conversely, OXPHOS genes exhibited a ‘High‐Low‐High’ trend (Figure 2J), decreasing during the intermediate phase and subsequently rebounding in the terminal LST state (State 3). This pattern suggests a potential stage‐specific metabolic reprogramming: OXPHOS signatures appear transiently suppressed during peak oncogenic signaling—which we hypothesize may support biosynthetic demands—before re‐elevating in the established lateral spreading phenotype, potentially to facilitate migration. In summary, LSTs exhibit distinct molecular features indicative of heightened malignant potential. These include the enrichment of CRC‐heredity–associated subpopulations, high expression of CRC risk genes, and distinct differentiation trajectories. These features are accompanied by elevated inferred CNV scores, which collectively suggest a coupling between these high‐CNV states and transcriptional programs associated with energy metabolism, epithelial barrier integrity, and extracellular matrix remodeling, potentially distinguishing LSTs from PAs and normal tissues.

### 2.3 Enhancement of Oxidative Phosphorylation Is a Key Event in LST Development

**FIGURE 3:**
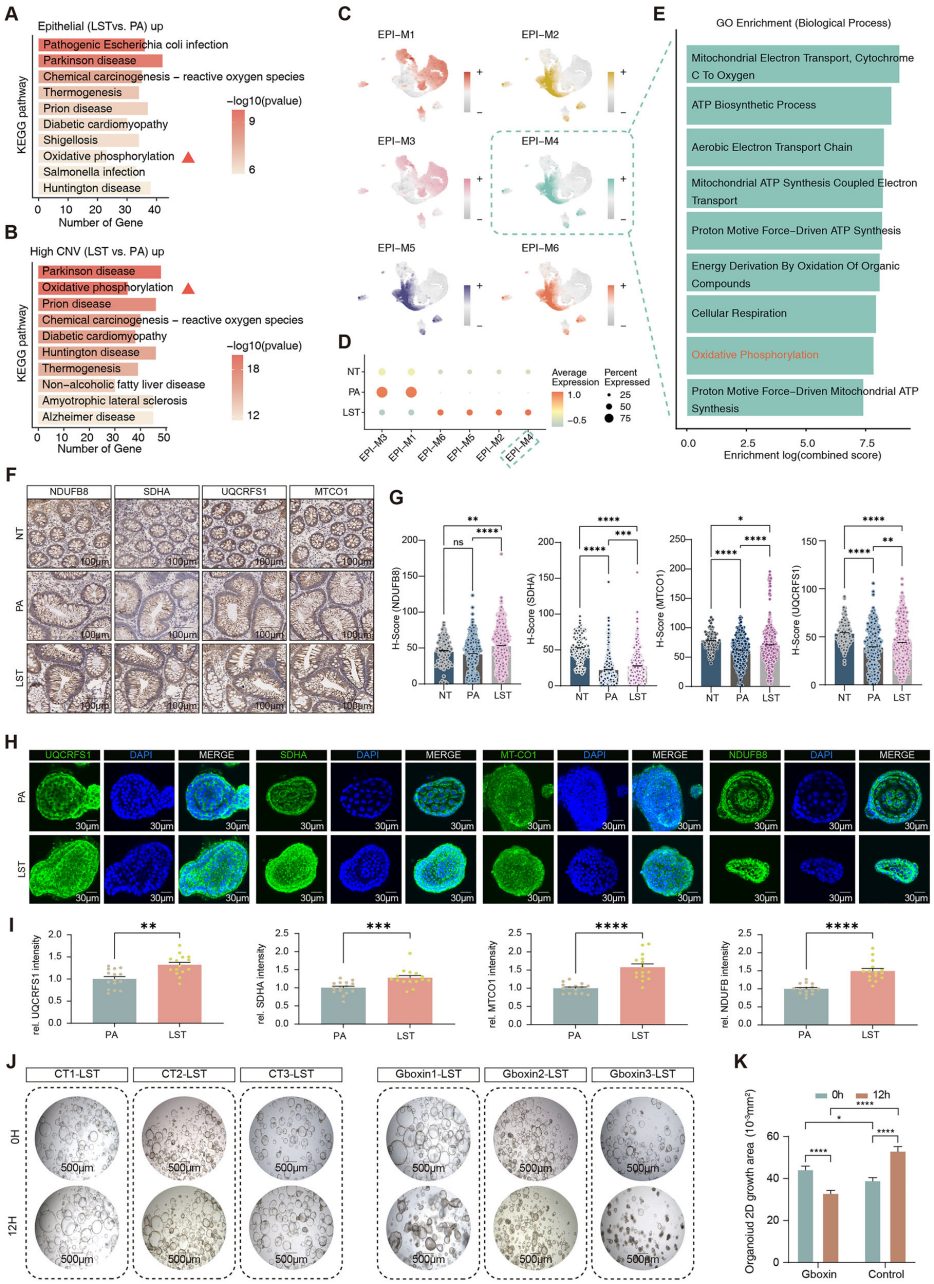
Enhancement of Oxidative Phosphorylation as a key event in LST progression. (A) KEGG analysis showing pathways upregulated in epithelial cells from LSTs compared with PAs. (B) KEGG analysis showing pathways upregulated in inferred high‐CNV epithelial cells from LSTs compared with their counterparts in PAs. (C) Feature plot of each co‐expression module, with cells colored according to the module's uniquely assigned color. (D) Dot plot showing the expression of the six identified epithelial modules across three groups. (E) GO enrichment analysis of terms associated with Epi‐Module 4. (F) IHC staining analysis of OXPHOS‐related genes in NT, PA, and LST tissue samples. (G) Bar plot presenting quantitative analysis results from (F) (n=15 for each group). *p* values were calculated using the one‐way ANOVA, followed by Tukey's post hoc test. (H) Immunofluorescence analysis of OXPHOS‐related genes in PA and LST organoids. (I) Bar plot displaying relative intensities of immunofluorescence signals in (H) (n=3 for each group). (J) Representative microscope bright‐field images (4× magnification) of LST organoids treated with the OXPHOS inhibitor Gboxin at 0 h and 12 h. (K) Bar plot depicting statistics analysis of changes in the organoid area from (J) (n=3 for each group). All *p* values were calculated using the Mann‐Whitney U test unless otherwise stated. *p < 0.05, **p < 0.01, ***p < 0.001, ****p < 0.0001.

To capture the key features of epithelial cells in LSTs versus PAs, we performed pathway enrichment and hdWGCNA analyses. Both KEGG and GO revealed upregulation of OXPHOS—a critical metabolic process implicated in tumorigenesis [26, 27] — in LSTs (Figure 3A,B; Figure S3A, B). Module EPI‐M4, characterized by OXPHOS and mitochondrial energy production, was highly expressed in LSTs (Figure 3C–E; Figure S3C), indicating that enhanced OXPHOS signatures represent a characteristic feature of LSTs.

To delineate the biological divergence between polypoid and laterally spreading trajectories, we performed functional enrichment analysis on the PA‐enriched epithelial clusters, EPI‐M1 and EPI‐M3. In contrast to the metabolic‐structural plasticity observed in LSTs, these PA‐specific populations exhibited molecular signatures characteristic of growth constraint and homeostasis. The EPI‐M1 cluster was significantly enriched for terms associated with structural rigidity and polarity maintenance, including “Contact inhibition”, “Establishment of centrosome localization”, and “Negative regulation of relaxation of muscle”, suggesting a retained cytoskeletal tension that potentially supports their protruding architecture (Figure S3D). Concurrently, the EPI‐M3 cluster displayed a distinct stress‐responsive profile, characterized by the upregulation of “Positive regulation of mitophagy” and “Negative regulation of growth” (Figure S3E). This active engagement of mitochondrial degradation and growth arrest pathways points to a potential metabolic braking mechanism in PAs that may counteracts the hyperactive OXPHOS state observed during lateral expansion.

To explore the relationship between inferred CNV burden and metabolism, we performed pseudo‐bulk analysis (Figure S3F), which showed a uniform upregulation of OXPHOS signatures in inferred CNV‐high versus CNV‐low epithelial populations across all LST patients. Conversely, this metabolic shift appeared highly variable among PA patients, further distinguishing the robust, LST‐specific metabolic reprogramming from the more stochastic patterns observed in conventional adenomas. To rigorously validate the stability of this metabolic divergence and mitigate potential biases from cohort imbalance, we performed a balanced subsampling test (n = 2 PA vs. n = 2 LST) restricted to the in‐house dataset. Across 10 independent stochastic iterations, mean OXPHOS expression remained consistently elevated in the LST group (Figure S3G). Furthermore, a systematic Leave‐One‐Donor‐Out (LODO) sensitivity analysis, conducted exclusively within this in‐house cohort, corroborated the robustness of these transcriptomic findings. Specifically, Gene Set Enrichment Analysis (GSEA) demonstrated that the positive enrichment of the OXPHOS signature in LST cells persisted across all donor‐exclusion permutations (Figure S3H). These analyses collectively indicate that the enhanced OXPHOS signature is a reproducible and consistent transcriptomic feature of LSTs, independent of specific donor combinations.

Clinical tissue samples and in vitro organoid experiments corroborated these transcriptomic findings, showing upregulated expression of key OXPHOS components—NDUFB8, SDHA, UQCRFS1, and MTCO1—in LSTs (Figure 3F–I). In LST‐derived organoids, treatment with the OXPHOS inhibitor Gboxin significantly reduced the organoid growth area (Figure 3J,K). In contrast, Gboxin treatment yielded no significant change in the growth area of PA‐derived organoids (Figure S3I,J), indicating that LST‐derived organoids exhibit a heightened and specific sensitivity to OXPHOS inhibition. Collectively, these results support the transcriptomic observations and indicate that OXPHOS upregulation represents a prominent and functionally relevant feature in LST development.

### 2.4 Enhanced Oxidative Phosphorylation Signatures are Coupled With Adhesion Molecule Downregulation in LSTs

**FIGURE 4:**
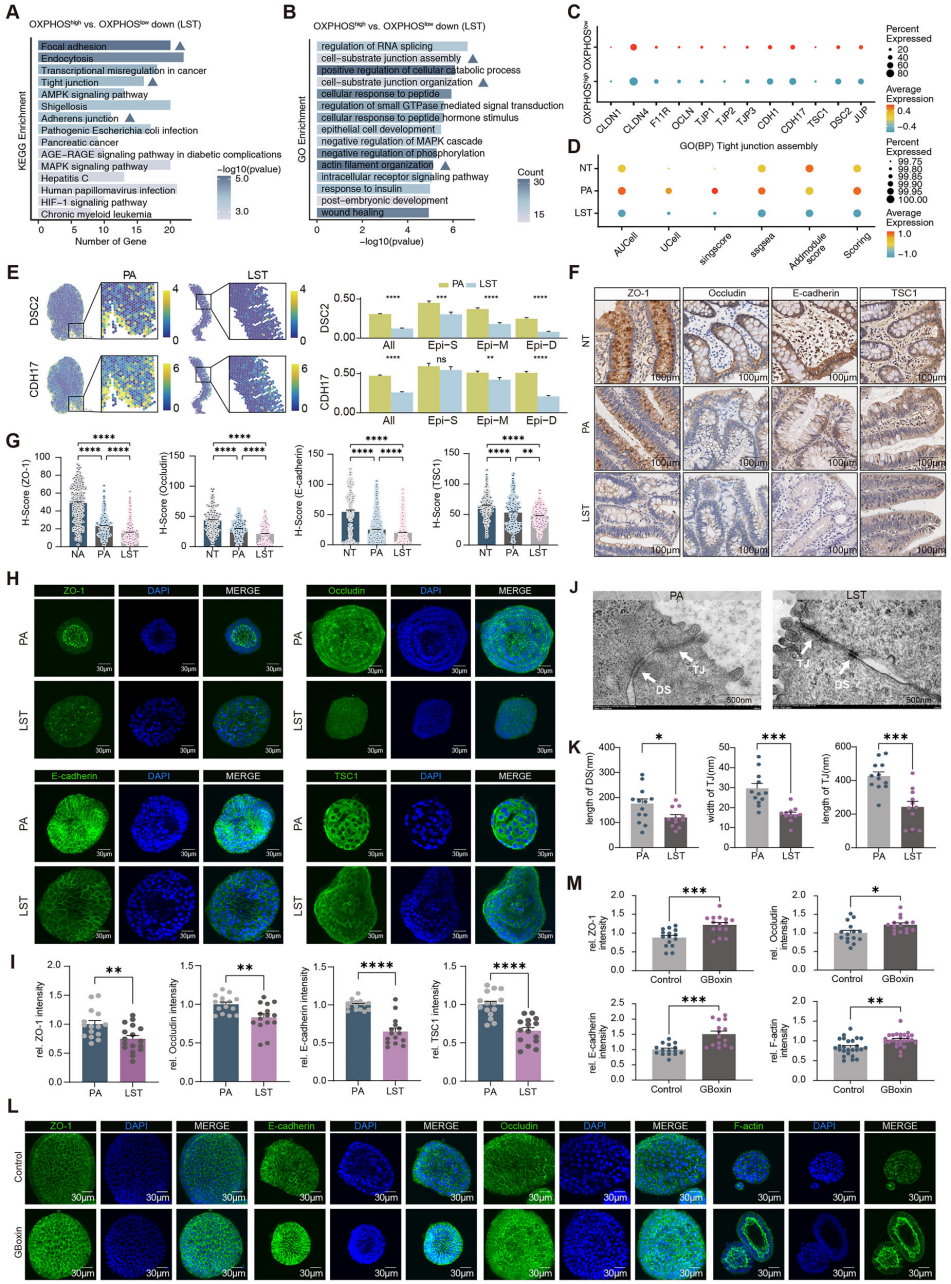
Enhanced Oxidative Phosphorylation Drives Adhesion Molecule Downregulation in LSTs. (A) KEGG analysis showing pathways downregulated in OXPHOS^high^ epithelial cells compared with OXPHOS^low^ epithelial cells within LSTs. (B) GO analysis showing pathways downregulated in OXPHOS^high^ epithelial cells compared with OXPHOS^low^ epithelial cells within LSTs. (C) Dot plot illustrating the expression of cell junction‐related genes in OXPHOS^high^ and OXPHOS^low^ cells. (D) Dot plot showing the signature scores for tight junction assembly in epithelial cells, calculated using different methods across the three groups. (E) Spatial plots depicting gene expression levels of cell junction molecules (DSC2 and CDH17) in PA and LST group, with statistical comparisons across distinct epithelial layers. (F) Representative IHC staining of adhesion moleculesOXPHOS‐related genes in mucosa tissue from NT, PA, and LST patients. (G) H‐score comparison of adhesion molecule expression among NT, PAs, and LSTs (n=15 for each group). *p* values were calculated using the one‐way ANOVA, followed by Tukey's post hoc test. (H) Representative immunofluorescence images of adhesion molecules in organoids derived from PAs and LSTs. (I) Quantitative analysis of adhesion molecule expression levels between PAs and LSTs (n=3 for each group). (J) Representative TEM images showing ultrastructural details of cell‐cell contacts in PA‐ and LST‐derived organoids. (K) Bar charts summarizing statistical analyses of DS length, TJ width, and TJ length (n=3 for each group). (L) Immunofluorescence analysis of tight junction markers (n=3) and cytoskeleton component F‐actin (n=5) in LST and PA organoids treated with Gboxin at 0 and 12 h. (M) Bar plot showing relative intensities of the immunofluorescence signals from (L). All *p* values were calculated using the Mann‐Whitney U test unless otherwise stated. *p < 0.05, **p < 0.01, ***p < 0.001, ****p < 0.0001.

Epithelial cells were subsequently stratified into OXPHOS^high^ and OXPHOS^low^ subpopulations based on the median OXPHOS signature score. Differential expression analysis between these two groups revealed downregulation of cell junction pathways—including focal adhesion, tight junction, adherens junction, and cell–substrate junction—in LST OXPHOS^high^ cells compared with its OXPHOS^low^ counterparts (Figure 4A,B). Pseudotime analysis further revealed an inverse expression trend between adhesion molecules and OXPHOS‐related genes in LST (Figure S4A, B) Notably, cell junction proteins (*CLDN1, CLDN4, F11R, OCLN, TJP1, TJP2, TJP3*), cadherins (*CDH1, CDH17*), desmosomal proteins (*DSC2*, *JUP*), and tight junction regulation gene (*TSC1*) were markedly downregulated in OXPHOS^high^ epithelial cells (Figure 4C). Signatures related to tight junction assembly, cell–cell junction organization, and maintenance of cell polarity consistently exhibited the lowest scores in LSTs (Figure 4D; Figure S4C, D). Collectively, these findings indicate that impaired intercellular adhesion is a hallmark of LSTs and may represent a key alteration closely coupled with elevated OXPHOS activity.

To determine whether this adhesion loss is a unique feature of LSTs, we performed a stratified analysis within PA tumors. We observed that PA OXPHOS^high^ cells indeed exhibited a downregulation of specific adhesion‐related pathways (Figure S4E, F); however, a critical divergence was observed in pathway hierarchy. In LST OXPHOS^high^ cells, ‘Focal adhesion’ emerged as the top‐ranked downregulated pathway, with ‘Tight junction’ also prominently enriched. In contrast, in PAs, the hierarchy was dominated by protein turnover pathways, specifically ‘Ubiquitin mediated proteolysis’, while ‘Focal adhesion’ ranked considerably lower in significance. Furthermore, we directly compared the OXPHOS^high^ subpopulations between the two groups. This analysis revealed that cell adhesion molecules were significantly downregulated in LSTs compared to their PAs counterparts (Figure S4G).

Crucially, to evaluate the robustness of this metabolic‐structural link, we performed a pseudo‐bulk analysis to quantify the fold change of cell adhesion molecules between OXPHOS^high^ and OXPHOS^low^ subpopulations across individual patients. This analysis uncovered a striking contrast in patient‐level consistency: adhesion molecules were stably and consistently downregulated in the OXPHOS^high^ compartment across all LST patients. In contrast, NT and PA samples exhibited significant inter‐patient heterogeneity, often displaying opposite trends or unchanged expression levels (Figure S4H, I). To further confirm that this inverse relationship is not driven by any single outlier patient, we extended our LODO analysis to the LST subpopulations. Consistent with the above findings, GSEA across all donor‐exclusion iterations revealed that the OXPHOS^high^ state stably maintained a significant negative enrichment for gene sets defining tight junctions, cell‐cell adhesion, and the establishment or maintenance of cytoskeleton polarity (Figure S4J). These findings underscore that the robust inverse coupling between enhanced OXPHOS signatures and the systematic downregulation of adhesion molecules is a highly specific hallmark of the LST trajectory.

To investigate the spatial and cellular heterogeneity in adhesion molecule downregulation, spatial transcriptomics (ST) was performed on lesion samples from one LST and one PA case. Based on gene expression and spatial clustering, tissue regions were classified into the superficial epithelial layer (Epi‐S), middle epithelial layer (Epi‐M), deep epithelial layer (Epi‐D), stromal regions (Stromal), and immune regions (Immune) (Figures S4K, L; S5A and Table S3A, B). Compared to PA, LST samples exhibited significant downregulation of adhesion‐related genes, including *CDH17* and *DSC2*, across the epithelial layers (Figure 4E). Consistently, immunohistochemical (IHC) analysis of clinical tissues demonstrated differential expression of adhesion molecules (e.g., ZO‐1, Occludin, E‐cadherin, TSC1, DSC2, DSG2) across the three groups (Figure 4F,G; Figure S5B). These observations were further validated by immunofluorescence staining in patient‐derived organoids (Figure 4H,I; Figure S5C,D). Ultrastructural analysis using transmission electron microscopy (TEM) revealed distinct morphological alterations indicative of structural disassembly in LST‐derived organoids. Rather than simple dimensional changes, we observed a marked atrophy of the junctional plaques themselves. Specifically, the electron‐dense structures of tight junctions (TJs) and desmosomes appeared significantly shorter in length and narrower in width compared with PA‐derived organoids (Figure 4J,K). This disintegration of the junctional scaffold coincided with the downregulation of the ‘Maintenance of Cell Polarity’ pathway (Figure S4D), suggesting that the attenuation of apical‐basal polarity parallels the structural collapse of these adhesion complexes while maintaining a cohesive cellular arrangement. Treatment with the OXPHOS inhibitor Gboxin significantly restored the expression of adhesion‐related molecules in LST‐derived organoids (Figure 4L,M; Figure S5E–G), supporting the notion that elevated OXPHOS activity negatively modulates adhesion molecule suppression in LSTs. Collectively, these results imply that reduced intercellular adhesion constitutes a crucial step in LST progression, which is tightly linked enhanced OXPHOS activity.

Given the well‐established interplay between adhesion molecules and cytoskeletal architecture [28], along with the reduced representation of cytoskeleton organization pathways in inferred high‐CNV LST cells, we investigated cytoskeletal dynamics. Downregulated genes were enriched in actin filament organization pathways (Figure 4B), suggesting a more relaxed cytoskeletal structure in OXPHOS^high^ cells. Consistently, significant actin fiber disassembly was observed in LST‐derived organoids (Figure S5H), whereas Gboxin treatment induced cytoskeletal aggregation (Figure 4L,M). Conversely, Cytochalasin D, a cytoskeleton‐depolymerizing agent, further suppressed adhesion molecule expression in LST organoids (Figure S5I–M). To ensure these effects were not due to acute toxicity or non‐specific junctional collapse, we treated LST‐derived organoids with a sub‐toxic concentration of Cyto D (100 nM) over a 24‐h time course. Quantitative analysis of the immunofluorescence revealed a progressive, stepwise decrease in F‐actin intensity (Figure S5N), confirming that a controlled, moderated reduction in cytoskeletal tension is capable of suppressing adhesion molecules. We further validated these findings at the protein level. Western blot (WB) analysis confirmed the downregulation of junctional proteins, an effect that was reversed upon treatment with Gboxin and further downregulated following CytoD treatment (Figure S5O–Q). Collectively, these results support a proposed model wherein elevated OXPHOS is coupled with cytoskeletal depolymerization, subsequently coinciding with the loss of adhesion molecules to potentially facilitate LST progression.

### 2.5 Molecular Interactions Between LST Epithelial Cells and Stromal Cells in the Matrix Microenvironment

To further delineate the biological landscape of LST epithelial cells, we utilized non‐negative matrix factorization (NMF) to identify interpretable gene‐program signatures that are consistently maintained within individual patients (Figure S6A). Among the 10 meta‐programs, MP1, MP2, and MP7 were associated with extracellular matrix (ECM), including ECM‐affiliated, Matrisome‐associated and ECM regulatory and degradation genes (Figure S6B–D), indicating a robust transcriptional interplay between epithelial cells and their stromal microenvironment in LSTs.

**FIGURE 5:**
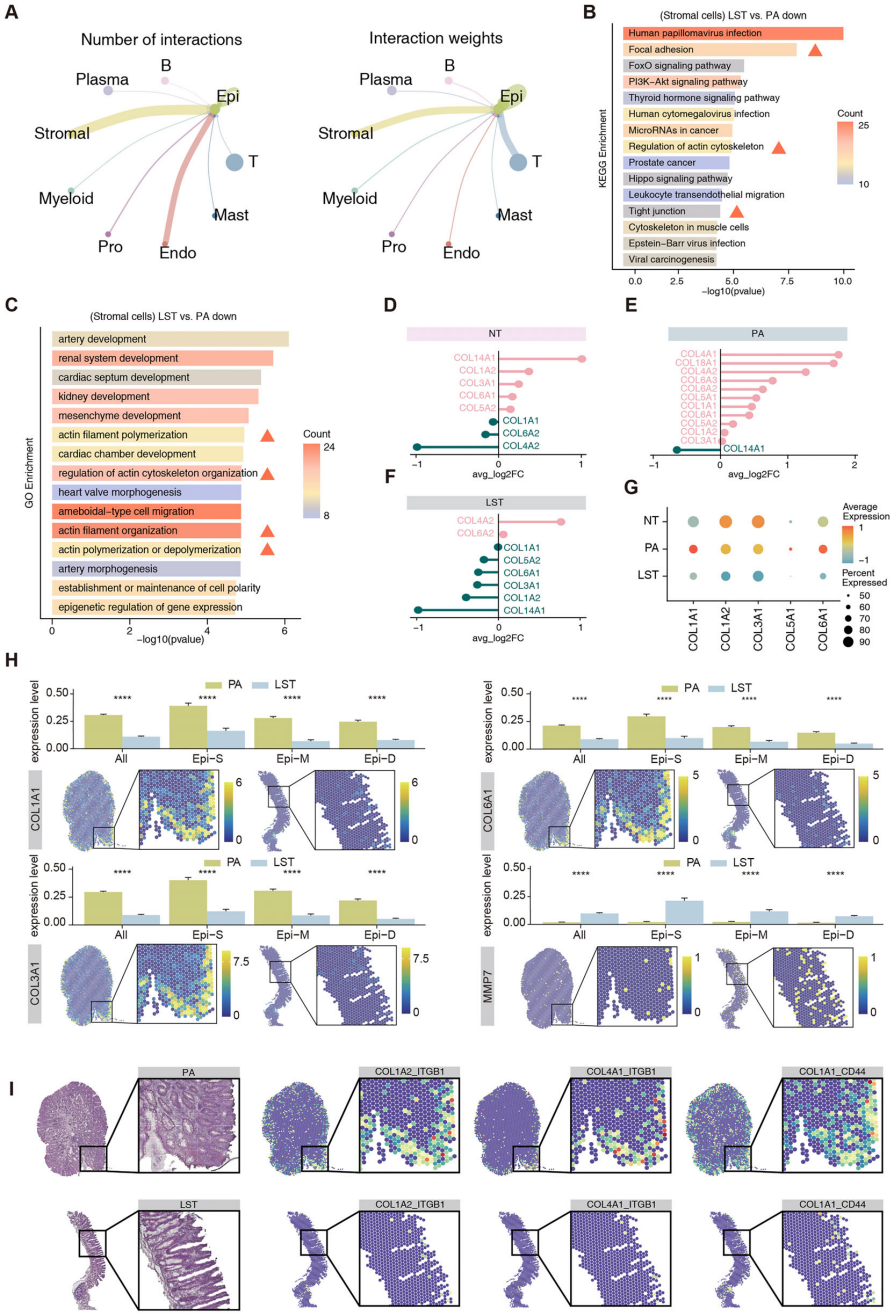
Characterization of molecular features in the stromal microenvironment. (A) Aggregated cell‐cell communication network illustrating the number of interactions (left) and the interaction weights (right), with epithelial cells designated as the target cluster. (B) KEGG analysis depicting pathways that are downregulated in stromal cells from LSTs relative to those from PAs. (C) GO analysis depicting pathways that are downregulated in stromal cells from LSTs relative to those from PAs. (D–F) Lollipop plots showing the fold changes of significantly differentially expressed collagen family genes across the three groups. (G) Dot plot depicting the expression levels of collagen family genes in stromal cells across three groups. (H) Spatial plots depicting the expression levels of collagen family genes (*COL1A1, COL6A1, COL3A1*) and MMP7 in PAs and LSTs, with statistical comparisons across different epithelial layers. (I) Spatial analysis using stLearn of specific ligand–receptor (LR) pairs in PA and LST sections, with interaction scores represented as –log10(p_adj) across all spatial spots.

Consistently, cellular interaction analysis revealed that stromal cells served as the dominant signaling source, exhibiting the highest number and strength of interactions with epithelial cells, suggesting a prominent communication node in LST progression (Figure 5A). Pathway enrichment analyses showed that LST stromal cells exhibited downregulation of focal adhesion, tight junction, and actin cytoskeleton or filament regulation pathways (Figure 5B,C). These patterns were also observed by Spatial transcriptomics‐based CellChat analysis, revealing reduced ECM‐associated signaling activity in LSTs compared with PAs (Figure S7A).

Further examination of DEGs across the three groups revealed that collagen expression was pervasively lower in LST stromal cells and their spatial landscape, a transcriptional feature typically associated with a softer, more compliant ECM (Figure 5D–H). To explore whether this soft matrix phenotype reflects intrinsic structural remodeling or active degradation, we integrated the secretory profiles of both stromal and epithelial compartments. Specifically, scRNA‐seq data showed downregulation of key genes involved in collagen production and matrix rigidification, such as LOXL1, SERPINH1, and SPARC, suggesting an attenuation of matrix cross‐linking and stabilization programs within the LST stroma (Figure S7B). This structural attenuation was further accompanied by the upregulation of factors promoting matrix hydration and tissue loosening, including HAS2 (Hyaluronan Synthase 2) and VCAN (Versican), alongside TNC (Tenascin C), which creates an anti‐adhesive intermediate matrix.

Critically, further analysis revealed that this predicted proteolytic remodeling is closely associated with the metabolic heterogenity of LST epithelial cells. Specifically, OXPHOS^high^ epithelial cells exhibited lower expression of collagen‐related molecules but higher levels of MMPs compared to their OXPHOS^low^ counterparts (Figure S7C). Among these, MMP2, MMP7, and MMP9 were specifically upregulated in the OXPHOS^high^ subpopulation of LSTs (Figure S7D). At the global cell level, the MMP family showed a comprehensive upregulation in LSTs compared to PAs (Figure S7E). Notably, the LST epithelial compartment specifically contributed to this process through the upregulation of MMP7 (Matrilysin), which we hypothesize may synergize with stromal‐derived proteases (e.g., CTSB, CTSL) to actively degrade residual structural barriers. Moreover, scRNA‐seq and spatial communication analysis both revealed stronger collagen‐related ligand–receptor interactions in PAs than in LSTs (Figure 5I; Figure S7F). Given this active “softening” of the niche, these observations lead us to hypothesize that LSTs may adopt a specialized migratory strategy tailored to a compliant environment. Interestingly, despite their aggressive spreading, LST epithelial cells exhibited a quiescent classical EMT program (Figure S7G, H). Consistent with a model of “Dual Plasticity,” our transcriptomic data showed a concurrent enrichment of Amoeboid, Actomyosin, and Protease‐independent gene programs (Figure S7I). This pattern points toward a potential hybrid migratory mode—potentially coordinated by the RHOA‐ROCK axis and contractile apparatus (MYH9/10, MYL9/12)—where LSTs might leverage enzymatic de‐adhesion to facilitate local spreading without undergoing a full mesenchymal transition.

In conclusion, these results suggest that a softer, more disorganized ECM and weakened collagen‐mediated stromal–epithelial crosstalk in LSTs may create a permissive mechanical niche that favors lateral expansion and malignant progression.

### 2.6 The Lateral Growth of Early Intestinal Tumors is Linked to a Softer Matrix Microenvironment

**FIGURE 6:**
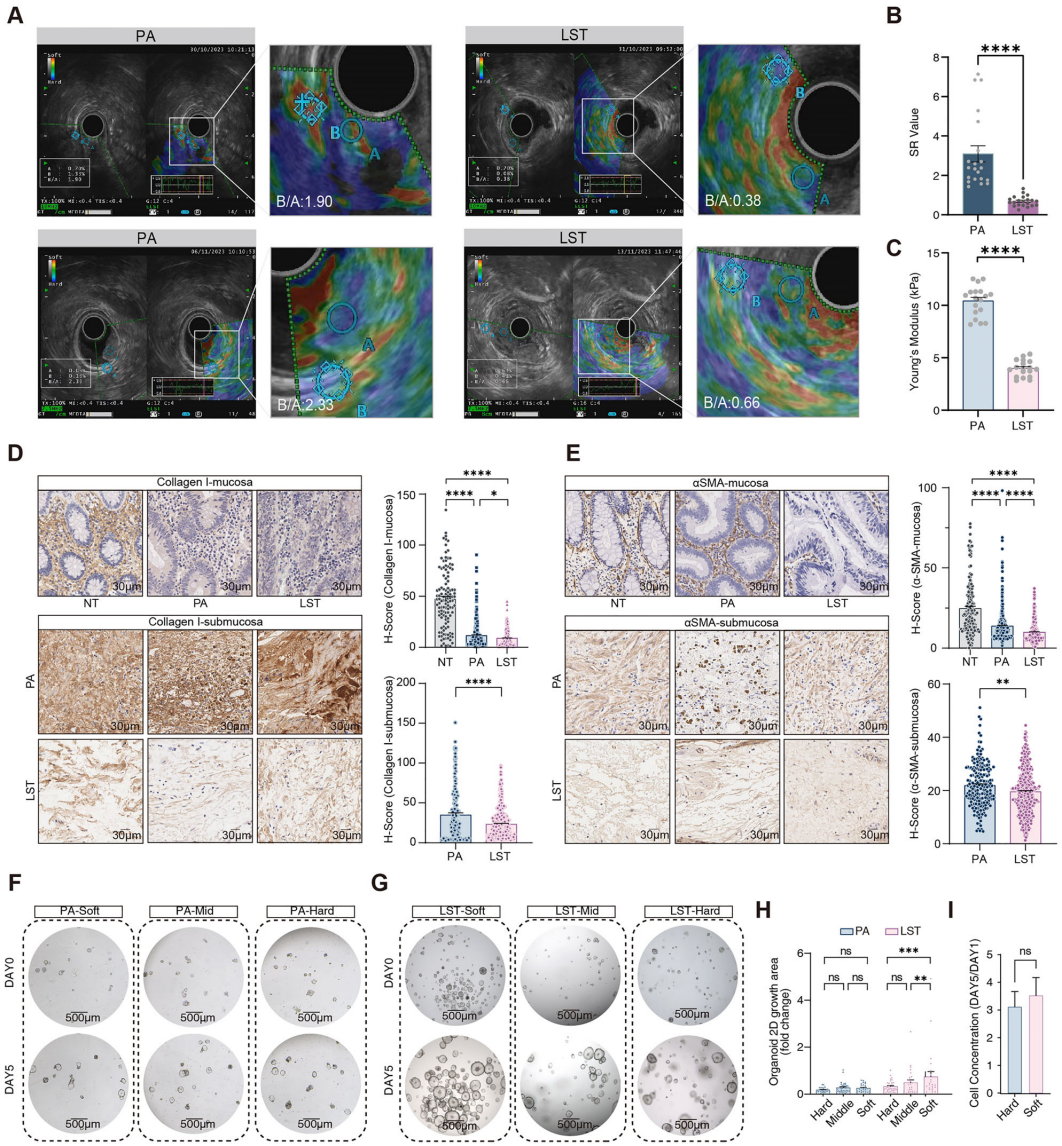
LSTs are located in the soft matrix microenvironment. (A) Representative endoscopic ultrasonography images demonstrating lesion sites (PA, n=7, left; LST, n=7, right) and adjacent regions within 5 cm. The B/A value indicates the elastic SR, reflecting tissue stiffness. (B) Bar plot displaying the comparison of elastic SR between LSTs and PAs assessed by endoscopic ultrasonography. (C) Bar plot displaying Young's modulus of laterally spreading tumor (LST) and protruding adenoma (PA) tissues measured by atomic force microscopy (AFM) (n=3 for each group). (D) Representative IHC staining images (left) and statistical analysis (right) of collagen I in mucosal and submucosal tissues from NT, PA, and LST samples (n=18 for each group). *p* values s were calculated using the one‐way ANOVA, followed by Tukey's post hoc test. (E) Representative IHC staining images (left) and statistics analysis (right) of α‐SMA in mucosal and submucosal tissues from NT, PA, and LST samples (n=14 for each group). *p* values were calculated using the one‐way ANOVA, followed by Tukey's post hoc test. (F) Representative bright‐field images (4× magnification) of PA organoids cultured on Matrigel of varying stiffness on day 1 and 5 (n=3 for each group). (G) Representative bright‐field images (4× magnification) of LST organoids cultured on Matrigel of varying stiffness on day 1 and 5 (n=3 for each group). (H) Bar plot showing the statistical analysis of organoid area fold changes (day 5/day 0), corresponding to (E, F) (n=3), one dot is for the area of one organoid. *p* values s were calculated using the one‐way ANOVA, followed by Tukey's post hoc test. (I) Cell proliferation analysis of LST organoids on substrates of varying stiffness. Organoids cultured on hard versus soft matrices were dissociated into single cells at days 1 and 5 for quantification. Data are presented as the ratio of day 5 to day 1 cell counts (mean ± SD, n = 3 independent experiments). All *p* values were calculated using the Mann‐Whitney U test unless otherwise stated. ns, no significance, *p < 0.05, **p < 0.01, ***p < 0.001, ****p < 0.0001.

To further evaluate our proposed model, we assessed the strain ratio (SR) of lesions versus adjacent normal tissues in patients with PAs and LSTs using endoscopic ultrasound elastography, where SR is positively correlated with matrix stiffness [29]. Consistent with our expectations, LST lesions exhibited significantly lower SR values, indicating a softer matrix microenvironment (Figure 6A,B). To directly quantify the mechanical properties at the tissue level, we performed atomic force microscopy (AFM) on clinical specimens. The Young's modulus values of LST tissues were significantly lower than those of PA tissues, further supporting the presence of a markedly softer microenvironment (Figure 6C). IHC analysis further revealed reduced expression of collagen type I (COLLAGEN I) and α‐smooth muscle actin (α‐SMA), markers of myofibroblasts, in the mucosal and submucosal compartments of LSTs compared with PAs (Figure 6D,E).

To functionally explore the association between a softer matrix environment and LST development, we cultured patient‐derived organoids on 2D matrices of varying stiffness to mimic the mechanical environment of the intestinal wall. LST‐derived organoids exhibited significantly enhanced lateral expansion on soft substrates, whereas PA‐derived organoids showed no significant stiffness‐responsive growth differences (Figure 6F–H). Importantly, quantitative assessment of cell concentration revealed no significant differences between the soft and hard matrix conditions (Figure 6I). This observation indicates that the enhanced lateral area observed on soft substrates primarily reflects morphological spreading and cellular rearrangement, rather than a mere increase in cellular proliferation. These results suggest that the spreading morphology of LST organoids is sensitive to matrix stiffness, consistent with the softer microenvironment observed in patient tissues. Collectively, these findings suggest that LSTs possess inherently softer mechanical properties and that a softer and compliant extracellular matrix may serve as a pliable physical substrate conducive to LST expansion and progression.

### 2.7 ENTPD1–ADORA2B Signaling Modulates LST Progression in Response to Matrix Stiffness

**FIGURE 7:**
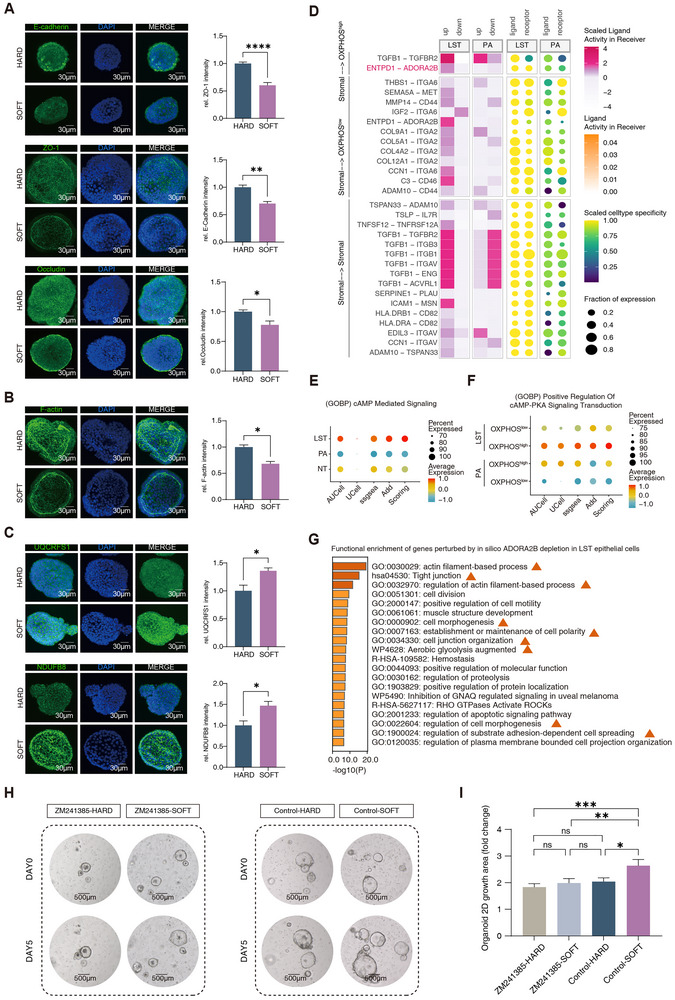
Soft matrix microenvironment promotes LST development through ENTPD1‐ADORA2B. (A,B) Immunofluorescence analysis of tight junction and cytoskeletal molecules in LST organoids cultured under hard and soft matrix conditions (left) and bar plot showing relative intensities of the immunofluorescence signals (right) (n=3 for each group). (C) Immunofluorescence analysis of OXPHOS‐related genes in LST organoids cultured under hard and soft matrix conditions and bar plot showing relative intensities of the immunofluorescence signals (n=3 for each group). (D) Bubble plots showing the top interactions among OXPHOS^high^, OXPHOS^low^, and stromal cells specific for LSTs with stromal cells as senders. (E) Dot plot illustrating the gene set enrichment scores for ‘cAMP‐mediated signaling’ (GOBP) across LST, PA, and NT epithelial groups. Scores were calculated using four independent algorithms (AUCell, UCell, ssGSEA, and AddModuleScore), with the ‘Scoring’ column representing the integrated sum of these four methodologies. (F) Dot plot illustrating the gene set enrichment scores for ‘positive regulation of cAMP‐PKA signaling transduction’ (GOBP) in OXPHOS‐high and OXPHOS‐low subpopulations within LST and PA groups. Scores were calculated using four independent algorithms (AUCell, UCell, ssGSEA, and AddModuleScore), with the ‘Scoring’ column representing the integrated sum of these four methodologies.″ (G) Metascape bar graph illustrating the biological pathways enriched among the top 300 genes (excluding ribosomal genes) affected by the in silico depletion of ADORA2B in LST epithelial cells. (H) Representative bright‐field images (4× magnification) of LST organoids treated with the ADORA2B inhibitor ZM241385 on day 0 and 5, cultured on Matrigel of varying stiffness (n=3 for each group). (I) Bar plot showing the statistical analysis of organoid area fold changes (day 5/day 0), corresponding to (H). All *p* values were calculated using the Mann‐Whitney U test. *p < 0.05, **p < 0.01, ***p < 0.001, ****p < 0.0001.

To further explore whether and how a soft matrix microenvironment interacts with key pathways implicated in LST progression, we cultured LST‐derived organoids on substrates of varying stiffnesses. Organoids grown on soft matrices exhibited pronounced downregulation of cell junctions and cytoskeletal proteins, accompanied by upregulation of OXPHOS components (Figure 7A–C). To identify potential mechanosensitive surface receptors associated with these changes, we performed cell–cell communication analysis using MultiNicheNet, which enabled an elaborate inference of ligand–receptor interactions and downstream targets between stromal cells and OXPHOS^high^ or OXPHOS^low^ epithelial cells in both PAs and LSTs. In the LSTs, stromal cells primarily interacted with OXPHOS^high^ cells through the *ENTPD1*‐*ADORA2B* axis, showing the highest ligand and receptor activity with tissue specificity. In contrast, stromal interactions with OXPHOS^low^ cells were predominantly mediated by collagen‐related signaling and collagen‐associated interactions were more active in PAs (Figure 7D; Figure S8A–C).

Given that ENTPD1 and ADORA2B cooperatively regulate extracellular adenosine signaling and have been implicated in tumor progression [30], and that enhanced adenosine metabolism may support mitochondrial OXPHOS [31], we next tested the functional relevance of this pathway. Consistent with the specific engagement of the ENTPD1‐ADORA2B axis in LSTs, downstream pathway analysis revealed that cAMP signaling and adenosine metabolism—canonical cascades activated by ADORA2B—exhibited the highest activity levels in LSTs compared to other groups (Figure 7E,F; Figure S8D).

To further elucidate the downstream effects of ADORA2B signaling, we performed in silico knockout of ADORA2B in LST epithelial cells. The resulting perturbed genes were enriched in pathways related to cell‐cell junctions, the actin cytoskeleton, cell morphology, and metabolic regulation (Figure 7G; Figure S8E). To further characterize the intrinsic transcriptomic features of ADORA2B‐expressing cells, we stratified LST epithelial cells into ADORA2B^+^ and ADORA2B^−^ subpopulations. KEGG enrichment analysis of differentially expressed genes revealed that the ADORA2B^+^ subpopulation exhibited the most significant upregulation of OXPHOS, while classic adherens junction pathways were markedly downregulated (Figure S8F,G). We next treated LST‐derived organoids with ZM241385, a selective ADORA2B antagonist. Under soft matrix conditions, ADORA2B inhibition significantly reduced organoid lateral expansion compared with the control, and attenuated the stiffness‐responsive growth advantage (Figure 7H,I). Consistently, immunofluorescence analysis revealed that ADORA2B inhibition in LST‐derived organoids led to a marked upregulation of junctional proteins (E‐cadherin, ZO‐1) and cytoskeletal components (F‐actin) (Figure S8H,I). Collectively, these findings indicate that ADORA2B signaling is a critical component of matrix‐responsive expansion, likely supporting a spread cellular state linked to altered OXPHOS, cell junctions and cytoskeletal organization.

Together, these findings suggest that LSTs exhibit reduced collagen‐mediated stromal–epithelial interactions compared with PAs. Instead, a softer extracellular matrix coincides with enhanced OXPHOS activity and weakened cell–cell adhesion, phenotypes that are closely coupled with the ENTPD1‐ADORA2B signaling axis to support LST progression

## 3 Discussion

Compared with conventional PAs, LSTs pose greater clinical challenges, including limited sensitivity in early detection, higher recurrence rates, and increased malignant potential, ultimately portending poorer patient outcomes. These unmet clinical needs highlight the urgency to elucidate the biological mechanisms that govern LST development and progression. In this study, by integrating single‐cell transcriptomics, spatial transcriptomics, and patient‐derived organoid models, we provide a comprehensive cellular and molecular atlas of LSTs, which, to our knowledge, is the first multi‐omic atlas delineating LST biology. Our analyses revealed that LSTs acquire a more malignant molecular phenotype with higher CNV levels, in part reflecting their stronger genetic risk enrichment for CRC. Functional assays further demonstrate that elevated OXPHOS is closely linked to the downregulation of epithelial adhesion molecules, thereby facilitating LST progression. Moreover, cell–cell interaction analyses and organoid‐based experiments support a model in which a softened ECM microenvironment serves as a key niche accommodating the characteristic lateral expansion and malignant evolution of LSTs.

Although LSTs are associated with a higher risk of submucosal infiltration and malignant transformation, current knowledge predominantly stems from preliminary clinical observations or cell line–based studies. These approaches lack systematic molecular characterization of in situ lesions and fail to accurately mimic tumor growth patterns within the intestinal wall. In particular, the contribution of the local microenvironment to the lateral spreading phenotype of LSTs remains poorly defined. Here, by integrating single‐cell and spatial transcriptomic datasets, we comprehensively delineate the multicellular ecosystem of LST lesions and identify critical intracellular and microenvironmental pathways implicated in their progression. Furthermore, to better recapitulate the lateral spreading morphology, we cultured patient‐derived organoids on Matrigel‐based substrates spanning a range of stiffnesses. This in vitro mechanical modeling revealed that LST‐derived organoids display accelerated and expansive lateral growth compared with PAs, including increased growth rates and enlarged transverse areas.

While heightened malignancy is a defining feature of LST epithelial cells, intrinsic properties alone cannot fully account for their distinctive lateral spreading behavior. To elucidate the underlying mechanisms, we performed in‐depth molecular analyses and confirmed a pronounced increase in OXPHOS activity within LST epithelial cells—consistent with prior studies demonstrating that elevated OXPHOS is associated with malignant progression and invasion across various tumor types [32, 33, 34]. Notably, this metabolic shift in LSTs coincided with substantial suppression of tight junction proteins, E‐cadherin, and desmosomal components. Such loss of adhesion has been increasingly recognized as a central mechanism associated with tumor cells acquiring invasive phenotypes and infiltrating surrounding tissues [35, 36].

Our trajectory analysis further refines the understanding of this metabolic plasticity. The observed ‘High‐Low‐High’ OXPHOS pattern, inversely mirroring the CRC risk gene trajectory, suggests a stage‐specific metabolic switch. We propose that the transient suppression of OXPHOS during the intermediate transition phase may support the rapid biosynthetic accumulation required for neoplastic transformation. Crucially, the subsequent metabolic rebound in the terminal state indicates that established LST cells re‐engage mitochondrial respiration. This reactivation likely supplies the ATP required to sustain cytoskeletal remodeling and lateral migration, suggesting a functional coupling between metabolic reprogramming and the physical demands of the spreading phenotype.

The observation that OXPHOS protein levels in LSTs are only modestly elevated compared to PAs—and sometimes lower than in physiological normal epithelium—should be interpreted through the lens of this dynamic plasticity. Since normal colonocytes inherently maintain high metabolic activity for barrier function, the ‘modest increase’ in LSTs represents a vital adaptive retention or escape from the metabolic suppression typical of conventional adenomas, rather than a supra‐physiological overexpression. Furthermore, the ‘High‐Low‐High’ trajectory implies that LST cells undergo functional metabolic oscillations. Therefore, static immunohistochemical assessment inevitably captures a heterogeneous snapshot of cells in varying metabolic phases, potentially diluting the perceived magnitude of upregulation. Crucially, however, our data demonstrates that despite this temporal dynamism, the relationship between this metabolic program and cellular phenotype remains highly robust. As revealed by our patient‐stratified analysis, the coupling between metabolic resurgence and structural disassembly is exceptionally consistent in LSTs. Whenever LST cells enter the high‐OXPHOS state (resembling the terminal trajectory), they consistently exhibit the adhesion‐downregulation program. This stands in sharp contrast to PAs, where high metabolic states do not consistently coincide with structural loss. Thus, it is not the absolute magnitude of the metabolic signal, but the robust, disease‐specific coupling of ‘OXPHOS‐recovery to Adhesion‐loss’ that strongly characterizes the aggressive lateral spreading phenotype.

To further delineate the biological divergence between these growth patterns, we also characterized the PA‐enriched epithelial populations (EPI‐M1 and EPI‐M3), which offered a striking mechanistic counterpoint. In contrast to the metabolic‐structural plasticity observed in LSTs, these PA‐specific clusters exhibited molecular signatures of ‘growth constraint’ and ‘homeostasis.’ The enrichment of pathways such as ‘Contact inhibition’ and ‘Negative regulation of relaxation’ in EPI‐M1 implies a retained cytoskeletal tension that confines expansion to the vertical axis. Concurrently, the upregulation of ‘Mitophagy’ and ‘Negative regulation of growth’ in EPI‐M3 points to a metabolic braking mechanism. This suggests that while PAs are confined by intrinsic structural rigidity and metabolic suppression, the LST phenotype is characterized precisely by the evasion of these checkpoints—relaxing the cytoskeleton and enhancing mitochondrial respiration to enable aggressive lateral expansion.

While reduced cell–cell adhesion facilitates migration, it alone is insufficient to explain the uniquely extensive lateral growth of LSTs observed clinically. From a biophysical perspective, beyond cell‐intrinsic alterations, a tumor‐supportive microenvironment—or ecological niche—appears to be critically involved in accommodating lateral expansion. Integrating clinical ultrasound elastography, single‐cell transcriptomics, and organoid‐based microenvironment modeling, we demonstrated that LST lesions possess a markedly softer extracellular matrix and reduced matrix deposition relative to both PAs and NTs. Moreover, LST‐derived organoids grown on soft matrices displayed significantly enhanced lateral expansion, faithfully recapitulating the in vivo spreading phenotype. These findings suggest that a stiffer peripheral environment may constrain the growth of conventional early tumors and promote a polypoid PA morphology, whereas a softer matrix, coupled with weakened cell–cell adhesion, is associated with the alleviation of spatial constraints and accommodates extensive lateral dissemination, ultimately giving rise to the LST phenotype.

Our study refines the understanding of this microenvironmental landscape by proposing that its characteristic ‘softness’ is not merely a passive feature but is synergistically maintained through a tripartite mechanism of ‘Impaired Stiffening, Active Softening, and Hyper‐Proteolysis.’ First, LST stromal cells exhibit significant attenuation in matrix rigidification, evidenced by the marked downregulation of cross‐linking and stabilizing enzymes [37] such as LOXL1, SERPINH1, and SPARC compared to the PA group. Second, this transcriptomic profile is compounded by the active secretion of ‘softening’ factors; LST stromal cells uniquely upregulate HAS2 (Hyaluronan Synthase 2) [38] and VCAN (Versican), which promote matrix hydration and tissue loosening, alongside TNC (Tenascin C) which creates an anti‐adhesive intermediate matrix. Third, and critically, this relaxed scaffold undergoes dynamic remodeling. Global differential expression analysis reveals a comprehensive upregulation of matrix metalloproteinases (MMPs)—including MMP1, MMP2, and MMP14—in LSTs compared to PAs. Notably, the LST epithelial compartment specifically contributes to this remodeling by upregulating MMP7, which synergizes with stromal‐derived proteases (e.g., CTSB, CTSL) to degrade residual structural barriers [39]. Consequently, this dynamically remodeled niche lowers the biophysical barrier for invasion, thereby creating a permissive environment for the cohesive, actomyosin‐driven motility observed in the laterally spreading trajectory.

Furthermore, our transcriptomic profiling supports a model of ‘Dual Plasticity’ underlying this dissemination, challenging the binary distinction between mesenchymal and amoeboid migration. We observed that LSTs uniquely co‐opt both proteolytic and contractile machineries to navigate their microenvironment. The global upregulation of MMPs, particularly MMP7 (Matrilysin), suggests an active capability for proteolytic remodeling—likely facilitating initial cell detachment via E‐cadherin cleavage and degrading stromal density to create a porous niche [40]. Crucially, this enzymatic activity functions synergistically with a robust Rho‐Actomyosin motor. We propose that while MMPs ‘soften’ the physical barriers and untether cells from the epithelial sheet, the upregulated actomyosin contractility provides the necessary cortical tension to support adaptive, squeeze‐like motility. This hybrid phenotype—combining enzymatic barrier‐breaching with structural deformability—enables LSTs to efficiently traverse the sub‐epithelial space, distinguishing their aggressive lateral spreading from the static, cohesive growth of polypoid adenomas [41].

Collectively, our findings advocate for a model of synergistic co‐evolution between tumor cell‐intrinsic programs and the mechanical microenvironment. While intrinsic alterations—characterized by a higher inferred CNV burden and oncogenic pathway activation (e.g., PI3K/EGFR)—serve as the fundamental components of neoplastic transformation and proliferation, they alone do not account for the macroscopic morphology. Instead, the mechanically ‘soft’ extracellular matrix functions as a critical physical modulator and permissive ecological niche. By spatiotemporally activating the mechanosensitive ENTPD1‐ADORA2B axis, this unique microenvironment effectively guides the intrinsically malignant cells toward a lateral spreading trajectory by relaxing cytoskeletal tension and reducing intercellular adhesion. Thus, the LST phenotype emerges not from the dominance of the TME over intrinsic biology, but from their integration, where the microenvironment unlocks and shapes the phenotypic plasticity of high‐risk neoplastic seeds.

Finally, we acknowledge several limitations of this study. First, the in vitro mechanical microenvironment was modeled using serially diluted Matrigel. We explicitly acknowledge that while this approach mimics the matrix‐depleted characteristics of the LST stroma, matrix dilution inherently alters confounding variables such as ligand density, pore architecture, and diffusion. Consequently, these in vitro PDO experiments cannot definitively isolate the causal contribution of stiffness per se from biochemical alterations. To rigorously disentangle mechanotransduction mechanisms, future studies must employ composition‐independent, mechanically tunable synthetic hydrogels with constant biochemical cues. In parallel, we must acknowledge that while our patient‐derived organoid (PDO) models provided valuable phenotypic observations regarding LST spreading behavior, 2D area readouts inherently possess limitations. Specifically, area expansion alone cannot definitively disentangle true invasive lateral extension from confounding variables such as enhanced cell survival, altered attachment efficiency, or physical flattening. Although our cellular quantification assays indicate that this morphological spreading is not primarily driven by increased proliferation, these observations should be cautiously interpreted as phenotypic correlates rather than definitive functional proof of invasion. Future investigations employing advanced orthogonal measurements—such as 3D volumetric reconstruction and high‐resolution live‐cell migration tracking—are warranted. Additionally, due to technical constraints, well‑established methods for directly assessing barrier permeability in organoids are currently lacking. Thus, developing assays to evaluate organoid barrier integrity and lateral invasion remains an important objective. The absence of robust protocols for generating normal intestinal mucosal organoids has limited our ability to perform comparative functional analyses; instead, we focused on direct functional comparisons between LST and PA organoids. Moreover, extracellular metabolites such as adenosine undergo rapid degradation. Future studies employing high‑resolution mass spectrometry for real‑time detection could help clarify the biochemical relationships within the ATP‑adenosine‑cAMP cascade, thereby advancing our understanding of the mechanochemical mechanisms underlying LST progression. Finally, although patient‑derived models provide compelling human‑relevant evidence, the use of engineered animal models with tunable extracellular matrix properties in future research will allow more comprehensive validation of the therapeutic potential of the ENTPD1‑ADORA2B axis.

In summary, our study reveals that LSTs are characterized by heightened malignancy, accompanied by a distinctive mechanical and molecular landscape marked by a softer ECM and enhanced OXPHOS. These features coincide with the suppression of epithelial adhesion molecules and support lateral tumor progression. Our work highlights a previously underappreciated mechano‐metabolic axis—linking matrix stiffness, mitochondrial metabolism, and cell adhesion—as a critical element in LST development. Importantly, targeting this axis by modulating ECM mechanics or OXPHOS activity may offer promising strategies for early detection and risk stratification of patients with LSTs.

## 4 Materials and Methods

### 4.1 Clinical Data Collection

Clinical data were gathered from four centers encompassing a cohort of 3000 patients with PAs and 1488 patients with LSTs. Lesion sizes and the distribution of endoscopic findings, specifically PPV, JNET 2 B, JNET 3, and NICE 3 classifications, were determined based on both endoscopic evaluations and pathology reports. The pathology reports were also reviewed to identify the number of patients with high‐grade dysplasia, cancerous components, and submucosal invasion. Representative endoscopic and pathological images were included in the analyses. The baseline for clinical data can be found in Table S1. LST is an important factor causing submucosal invasion after univariate and multivariate logistic regression analyses. These analyses can be found in Table S5.

### 4.2 Sample Acquisition and Sequencing Procedures

Single‐cell transcriptome sequencing was performed on samples from seven LST patients, two PA patients, and two healthy controls. Spatial transcriptome sequencing was conducted on samples from one LST patient and one PA patient, respectively. All tissue samples were collected at the Department of Gastroenterology, Nanfang Hospital with ethical approval from the Ethics Committee of Nanfang Hospital, Southern Medical University (NFEC‐2024‐466). All clinical specimens in this study were obtained from living patients. LST samples were collected via endoscopic submucosal dissection (ESD) or endoscopic mucosal resection (EMR), PA samples were obtained through endoscopic snare polypectomy, and NT samples were acquired using endoscopic biopsy forceps. For all three groups, approximately 3–5 mm of fresh tissue was collected from each lesion. Immediately after resection, specimens were placed into a specialized preservation solution for single‐cell sample collection, maintained at 4°C, and subsequently transferred to the sequencing facility under a continuous cold‐chain condition. Additional tissue pieces were submitted to the pathology department for definitive histopathological diagnosis. All samples were derived from surgical/endoscopic biopsies rather than cadaveric sources; freezing, long‐term storage, and transport conditions followed standard clinical protocols, with no deviations expected to bias downstream analyses. After random sampling, statistical tests confirmed no significant differences in baseline characteristics between the groups.

### 4.3 Organoid Generation and Culture

Tumor samples were obtained from patients with LSTs and PAs during colonoscopy via endoscopic mucosal resection (EMR) or endoscopic submucosal dissection (ESD). The samples were washed five times with Dulbecco's phosphate‐buffered saline (PBS) containing antibiotics and then cut into 0.5 mm fragments using surgical scissors. These tissue fragments were digested in a solution containing 1.5 mg/mL hyaluronidase (Macklin, H810948‐100 mg) and 0.25 mg/mL type IV collagenase (Worthington, LS004188) at 37°C, rotating at 200 rpm for 40 min, and subsequently filtered through a 70 µm filter. Crypts and tissue fragments retained on the filter were collected and centrifuged at 600 g for 3 min, embedded in Matrigel (Corning, 356231), and cultured in colon cancer organoid medium (bioGenous, K2003‐HC‐500).

Organoid morphology was categorized based on the following optical features: spherical organoids consisting of a monolayer of intestinal epithelial cells surrounding a central cavity; budding organoids displaying surface protrusions with multiple small cavities; mature organoids exhibiting a well‐defined layered structure with villus‐like projections and crypt‐like domains in the epithelial layer.

### 4.4 Measurement of Elastic Strain Ratios Using Endoscopic Ultrasonography

Endoscopic ultrasound‐guided strain elastography (SE) operates by applying a mild external force to tissue, tracing the resulting tissue displacement through analysis of pairs of echo frames, and calculating strain from the spatial gradient of displacement. The strain ratio (SR) is then calculated by comparing the strain at the lesion site (point A) with that in a reference normal area (point B, located 5 cm away), represented by the B/A value [29, 42]. Stiffer lesions exhibit lower strain values, resulting in higher SR values.

All the patients underwent bowel ultrasonography after overnight fasting by means of a ultrasound processor (Olympus EU‐ME2 PREMIER PLUS; Olympus Medical Systems, Tokyo, Japan) under the circumferential view. SE examinations were performed after ultrasonography evaluation, whose principle and analysis were performed as above. All SE examinations were conducted by the same experienced endoscopist, with three repeated measurements per case. Data with poor reproducibility were excluded from the analysis. The endoscopist was blinded to the study hypothesis. In accordance with the elastography operating specification, the lesion confined to the mucosal layer was selected as point A, and the whole bowel wall of adjacent normal tissue was selected as point B. All lesions were confined to the rectum. All the SE analyses were performed with the software provided by the manufacturer (EVIS EUS; Olympus Medical Systems, Tokyo, Japan).

### 4.5 RNA‐Seq Library Preparation for 10× Genomics Single‐Cell 5′

Single‐cell suspensions were generated by enzymatic digestion with collagenase 1A (1 mg/mL) and DNase I (10 U/mL) with gentle rotation at 37°C for 30 min. Single‐cell 5′ gene expression libraries were prepared individually for each donor using the Chromium Single Cell 5′ Library kit following the manufacturer's protocol. Briefly, the single‐cell suspension was mixed with RT‐PCR master mix, loaded with nanoliter‐scale gel beads, and encapsulated in partitioning oil on a single cell 5′ chip. RNA transcripts from each cell were assigned unique barcodes. Following reverse transcription, the barcoded cDNAs were purified, amplified, end‐repaired, and ligated with Illumina adapters to generate a single multiplexed library. All libraries were sequenced on the Illumina Novaseq 6000 platform.

### 4.6 Preprocessing of Single‐Cell RNA‐Seq Data

The Cell Ranger v7.0.0 and v8.0.0 pipeline (10× Genomics) was used to demultiplex the cellular barcodes and align reads to the human transcriptome (GRCh38 build). Sequencing outputs from the seven subjects were normalized by sequencing depth and merged into a raw gene expression matrix (gene counts vs. cells). Using the Seurat v4.1.4 package in R [43], the unique molecular identifier (UMI) count matrix was converted into Seurat objects.

We also collected publicly available scRNA‐seq datasets from the NCBI Gene Expression Omnibus, including GSE161277 [44] (3 NTs and 4 PAs) and GSE261388 [45] (2 NTs and 1 PAs). Together with the datasets generated in this study, a total of 7 LSTs, 7 PAs, and 7 NTs were included for analysis. Detailed clinical characteristics, data provenance (including GEO accession numbers), and comprehensive donor‐level quality control (QC) metrics for each individual subject are summarized in Table S1. To ensure high‐fidelity downstream analyses, rigorous quality control (QC) was performed at the individual sample level. Cells were filtered by excluding those with fewer than 200 or more than 5000 detected genes, and cells with more than 15% UMIs originating from mitochondrial genes. The upper bound for detected genes served as an initial step to mitigate technical multiplets. To explicitly define our doublet exclusion strategy, we systematically filtered out cells exhibiting heuristic lineage conflicts, defined by the aberrant co‐expression of established multi‐lineage marker genes. Furthermore, to address potential ambient RNA contamination, we assessed background RNA levels; given the consistently low contamination profile across our dataset, we opted not to apply a mathematical ambient RNA correction step to avoid the potential over‐correction and loss of genuine biological signals. Following quality control, the filtered gene expression matrices were normalized using the *NormalizeData* function in Seurat.

### 4.7 Dimensionality Reduction, Batch‐Aware Integration, and Cell Clustering

After pre‐processing and quality control, the normalized gene expression matrices were subjected to feature selection to identify major cell clusters. We applied the *FindVariableFeatures* function in Seurat to select highly variable genes (HVGs). Principal component analysis (PCA) was then conducted on the merged global dataset. To rigorously correct for technical batch effects and inter‐individual variations, we employed the Harmony algorithm for dimensional reduction and integration, explicitly specifying the “donor” identifier as the batch covariate. Following this global, donor‐level batch correction, clustering was performed using the *FindClusters* function based on the first 10 Harmony‐corrected principal components with a resolution parameter set at 1.5. For visualization, t‐distributed stochastic neighbor embedding (t‐SNE) was generated using the RunTSNE function with default settings.

To further analyze functional cellular subsets within the epithelial compartment, we implemented a hierarchical, batch‐aware integration framework. Rather than simply inheriting the global batch‐corrected embeddings, the epithelial cell subset was extracted and re‐processed from the ground up. Specifically, we re‐identified epithelial‐specific HVGs, re‐scaled the expression matrix, performed a new PCA, and re‐applied Harmony integration exclusively within the epithelial population (again using the donor identifier as the batch covariate). This iterative, resolution‐specific approach minimizes residual noise from non‐epithelial lineages, avoids over‐smoothing, and ensures that batch correction is optimally calibrated to capture genuine biological heterogeneity within the epithelial compartment.

Following subset‐specific Harmony integration, epithelial sub‐clustering was achieved using the *FindClusters* function. To compare the composition and transcriptional profiles across the NT and disease (PAs and LSTs) groups, differentially expressed genes (DEGs) and specific marker genes for each subset were identified using the *FindAllMarkers* function in Seurat. Epithelial cell transcriptomic signatures were further annotated and explored using the CellMarker2.0 database [46]. Further descriptions of these subsets and their marker genes are detailed in the figures and main text.

### 4.8 Identification of DEGs and Pathway Analysis

DEGs across cellular subsets and tissue groups were identified using the *FindMarkers* or *FindAllMarkers* functions in Seurat. The biological functions and signaling pathways associated with DEGs were assessed through GO and KEGG pathway enrichment analyses using the R packages “clusterProfiler” and “enrichplot.” Further enrichment analyses were conducted using the Metascape database [47]. Gene expression assessments across groups were performed using the “scRNAtoolVis” package (https://github.com/junjunlab/scRNAtoolVis).

### 4.9 Gene Set Enrichment Analysis

Gene sets for signature scoring were sourced from the Molecular Signatures Database [48] to assess pathway activity. Five enrichment algorithms (AUCell, UCell, single‐score, ssGSEA, and AddModuleScore) were applied, followed by scaling and normalization. The total score is derived by summing the individual scores from each algorithm. Pearson correlation analysis was conducted to elucidate gene expression patterns.

### 4.10 Establishing the OXPHOS Score via Gene Set Scoring

To quantify oxidative phosphorylation (OXPHOS) activity, we utilized the KEGG_OXIDATIVE_PHOSPHORYLATION.v2023.2.Hs.gmt gene set (Online Supplemental table S4), which included 132 genes related to oxidative phosphorylation. Using the Seurat AddModuleScore function, the OXPHOS score was assigned to each cell. Epithelial cells were subsequently categorized into OXPHOS^high^ and OXPHOS^low^ groups, based on the median OXPHOS score.

### 4.11 Robustness Validation and Sensitivity Analyses

To ensure the reliability of our findings and confirm that the identified biological programs were not driven by individual donor idiosyncrasies or sample size imbalances, we performed extensive sensitivity analyses exclusively within our in‐house dataset (n = 7 LSTs and n = 2 PAs).

First, we implemented a donor‐level permutation subsampling strategy to verify the stability of the LST‐associated OXPHOS signature. In each of the 10 independent iterations, we randomly subsampled two donors from the LST cohort to be compared against the two PA donors. To ensure an unbiased assessment, average expression levels were calculated using the raw RNA assay, accessing the full depth of the transcriptome. The consistent metabolic divergence observed across all iterations—quantified by the mean expression of leading‐edge OXPHOS genes—definitively confirmed that the metabolic upregulation in LSTs is a robust feature independent of donor‐specific cell counts.

Second, we conducted a Leave‐One‐Donor‐Out (LODO) sensitivity analysis using the *Seurat* and *clusterProfiler* R packages. This iterative framework was applied to evaluate both the global OXPHOS shift between lineages and the functional coupling between OXPHOS activity and cytoskeletal/adhesion programs within the LST cohort. For each iteration, transcriptomic data from a single designated in‐house donor were computationally excluded. Differential expression analysis was then re‐performed on the remaining subset (logfc.threshold = 0, min.pct = 0.05), followed by GSEA on the descendingly ranked gene list. For intra‐LST validation, cells were stratified into OXPHOS^high^ and OXPHOS^low^ groups using *AddModuleScore* prior to each LODO iteration. The consistent directionality and statistical significance of the enrichment scores (NES) across all LODO iterations across our internal dataset confirmed the exceptional stability of the identified mechano‐metabolic axis.

### 4.12 Differential Abundance Analysis

Milo, a graph‐based differential abundance framework that quantifies shifts in cellular composition by testing discrete neighborhoods across the single‐cell manifold [49], was employed to robustly identify epithelial subpopulations exhibiting altered abundance between LSTs and PAs.

### 4.13 Evaluation of Single‐Cell Copy Number Variation

To differentiate malignant cells from normal cells, copy number variation (CNV) levels were assessed using InferCNV (version 1.14.2). We stratified epithelial cells into low, median, and high CNV groups. Briefly, CNV scores were computed for each cell based on the InferCNV results. Cells were then ranked by CNVscore, and grouped according to quartile distribution: the lowest 25% were defined as the low CNV group, the highest 25% as the high CNV group, and the remaining 50% as the median CNV group.

### 4.14 Assessment of Tumor‐Related Signaling Pathway Activity

Activity scores for tumor‐related signaling pathways were computed with the R package “PROGENy” [50].

### 4.15 Non‐Negative Matrix Factorization Analysis

NMF was performed using R package “GeneNMF” (version 0.6.2) [51]. Gene expression matrices were decomposed into distinct metaprograms (MPs) across LST samples to identify the underlying transcriptional patterns. MPs with consistent patterns across samples were retained for downstream analyses including functional enrichment and correlation assessments. Ten MPs were identified in the present study.

### 4.16 Weighted Gene Correlation Network Analysis of Bulk RNA‐Seq and scRNA Data

For network analysis, traditional WGCNA [52] was used for bulk RNA‐seq data, and high‐dimensional WGCNA (hdWGCNA) [53] was applied to scRNA‐seq data. Both analyses used the “WGCNA” (v1.72‐1) and “hdWGCNA” (v0.3.03) R packages to identify the key modules relevant to PAs and LSTs. An optimal soft threshold was chosen to convert the correlation matrix into an adjacency matrix.

For bulk RNA‐seq, three modules showed significant correlations. Genes from the MEturquoise module positively associated with PAs and the MEbrown module associated with LSTs were selected for functional enrichment analysis using Metascape. In the scRNA‐seq data, genes expressed in at least 5% of epithelial cells were used to construct the hdWGCNA object, which was subsequently converted into a Metacells object. Following the hdWGCNA standard pipeline, a correlation network was generated using a soft power threshold of 5. Module eigengenes (MEs), representing the gene expression profiles of co‐expression modules, were used for further analyses.

### 4.17 scPagwas Analysis

scPagwas is a computational framework that integrates single‐cell transcriptomes with genome‐wide association study (GWAS) summary statistics to identify cell types or cellular programs enriched for genetic risk variants [54]. In this study, we obtained CRC GWAS summary data from the OpenGWAS database (https://opengwas.io/datasets/). Specifically, we selected an East Asian cohort dataset (OpenGWAS ID: bbj‐a‐107) for downstream scPagwas analysis.

### 4.18 Cell Trajectory Inference

Trajectory inference (TI) is instrumental in understanding cell development and the associated biological processes. The Monocle algorithm (version 2.26.0) [55] was used to trace the developmental origins of epithelial cells. Highly variable genes were selected based on the default parameters, followed by the DDR Tree method for dimensionality reduction.

### 4.19 Cell‐Cell Communication Analysis

Intercellular interaction analysis was conducted using multiple packages with standardized parameters. CellChat [56] was applied to both scRNA‐seq and ST data, whereas the MultiNicheNet package (version 2.0.0) [57] was used to identify cross‐patient and cross‐tissue interactions based on ligand‐receptor signaling pathways. Each tissue group was compared with the others to identify group‐specific cell‐cell communications, following the default MultiNicheNet pipeline for comprehensive interaction analysis.

### 4.20 Spatial Cell‐Cell Interaction Analysis

Cell‐cell interaction (CCI) analysis was performed using StLearn (v0.4.0) [58]. Tissue regions with high cellular diversity and significant ligand‐receptor co‐expression were identified as hotspots with elevated CCI activity. The CCI score was calculated for each spot to quantify CCI levels. Our analysis focused specifically on spatial ligand‐receptor interactions linked to extracellular matrix (ECM) dynamics.

### 4.21 Virtual Knockout Analysis

To evaluate the transcriptomic regulatory influence of ADORA2B on LST epithelial cells, we conducted in silico virtual knockout (vKO) simulations using the R package scTenifoldKnk (v1.0.1) [59]. Briefly, the filtered raw count matrix was used as input to construct a reference gene regulatory network (GRN) via the scTenifoldNet pipeline. A vKO network was subsequently generated by zeroing out the out‐degree edge weights of the target gene (ADORA2B). The structural deviations between the reference and vKO networks were quantified using tensor decomposition‐based manifold alignment. We ranked all genes by their differential regulation fold change (FC) scores, which reflect the magnitude of their structural displacement in the manifold. The top 300 genes with the highest FC values were identified as differentially regulated genes (DRGs) and prioritized for downstream functional enrichment analysis to elucidate the biological pathways modulated by ADORA2B.

### 4.22 Analysis of Bulk RNA Data

Two bulk RNA datasets were analyzed, including 61 PA samples and 11 LST samples. The “sva” algorithm was applied to correct for batch effects across datasets. Dimensionality reduction and visualization were achieved through PCA to assess the effectiveness of batch effect correction for downstream analyses. The datasets supporting this study are available from the NCBI Gene Expression Omnibus repository (GSE233602 for PAs and GSE77635 for LSTs).

### 4.23 Spatial Transcriptomic Sequencing

Tumor tissues excised during endoscopic surgery were rinsed with precooled RNase‐free PBS, sectioned, and embedded in OCT compound. Samples were placed on dry ice and stored at −80°C. Ten‐micron‐thick sections were prepared and transferred to transcriptome chips (BMKMANU S1000) containing 1 to 8 identical 6.8 × 6.8 mm capture areas with 2 000 000 spots with barcoded primers. Primers were attached to the 5′ end of the slide and included a cleavage site, T7 promoter region, partial read 1 Illumina handle, unique spatial barcode for each spot, unique molecular identifier (UMI), and poly(dT)‐VN. The 2.5 µm‐diameter spots were arrayed in a hexagonal grid with a 4.8 µm center‐to‐center distance, creating a consistent pattern for spatial analysis. Tissue optimization, fixation, hematoxylin and eosin staining, and imaging were performed according to the manufacturer's protocol, followed by nuclear staining and imaging for cell segmentation. The samples underwent reverse transcription and spatial library preparation, with quality control and sequencing conducted on the Illumina NovaSeq 6000 platform to produce 150 bp paired‐end reads.

### 4.24 Spatial Transcriptomic Analysis

Upstream analysis was conducted using BSTMatrix (v2.3j) to generate the count matrix data for downstream analysis. Data processing involved normalization, clustering, and marker gene identification using the R package “Seurat” (v4.3.2). All mitochondrial genes were excluded from the analysis. Visualization was primarily conducted using Seurat and ggplot2.

### 4.25 Hematoxylin & Eosin (H&E) and PAS‐Alcian Blue (PAS‐AB) Staining

Organoids were collected in precooled PBS and incubated at 4°C for 30 min to completely dissolve Matrigel. The samples were then washed twice with PBS, fixed in 4% paraformaldehyde for 10 min, and embedded in paraffin. After deparaffinization, 5 µm sections were stained using a hematoxylin‐eosin kit (Solarbio, G1120) and PAS‐AB kit (Servicebio, G1049), according to the manufacturer's protocols. Glass slides were scanned with an Aperio CS2 digital scanner (Leica), and images were captured using the Image Viewer software.

### 4.26 Immunohistochemistry and Immunofluorescence Protocol

Paraffin‐embedded colonic biopsy sections were processed according to established protocols. For immunohistochemistry (IHC) staining, tissues were fixed in 10% formalin, embedded in paraffin, and sectioned at 5 µm thickness. Following deparaffinization and antigen retrieval, the sections were incubated in a blocking buffer (PBS containing 5% normal donkey or goat serum and 0.3% Triton X‐100) at room temperature for 1 h. Primary antibodies were applied in a wet chamber overnight at 4°C. Following PBS washes, sections were incubated with secondary antibodies for 45 min at 37°C. Visualization was performed using a hematoxylin and DAB colorimetric kit (Maxim, DAB4033). The slides were then scanned with an Aperio CS2 digital scanner (Leica) at 40× magnification, and 30 images were randomly captured from each slide using the Image Viewer for analysis with the IHC Profiler plugin in ImageJ. IHC scoring was calculated using the following formula: (high positive percentage × 3) + (positive percentage × 2) + (low positive percentage).

Primary antibodies for IHC included rabbit anti‐TSC1 (1:50; Beyotime, AF8256), mouse anti‐E‐cadherin (1:100, CST, 14472A), rabbit anti‐NDUFB8 (1:500, Abcam, ab192878), rabbit anti‐SDHA (1:500, Abcam, ab137040), rabbit anti‐UQCRFS1 (1:500, Abcam, ab191078), rabbit anti‐MTCO1 (1:250, Abcam, ab203912), rabbit anti‐collagen I (1:1500, Abcam, ab138492), and rabbit anti‐⍺‐SMA (1:3000, Proteintech, 14395‐1‐AP). The secondary antibodies used were goat anti‐rabbit secondary antibody (Abcam, ab205718, 1:2000) or goat anti‐mouse (Abcam, ab205719, 1:2000). All antibody dilutions were prepared using an antibody diluent (CST, 8112 L).

For immunofluorescence, organoids were collected from Matrigel and fixed in 4% paraformaldehyde for 10 min. The samples were permeabilized with a permeabilizing solution (Beyotime, P0095‐500 mL) for 15 min, followed by incubation in blocking solution (Beyotime, P0260) for another 15 min. The samples were then washed twice with phosphate‐buffered saline (PBS). Subsequently, the samples were incubated overnight with appropriately diluted primary antibodies, and visualization was performed using fluorescently labeled secondary antibodies. Slides were then incubated with Antifade Mounting Medium containing DAPI (Beyotime, P0131‐25 mL) for 10 min and imaged using a laser confocal microscope (Olympus FluoView FV1000) with a 60 × 1.42 oil immersion objective. Images were uniformly adjusted for brightness, opacity, contrast, and color balance for consistency. For quantitative analysis, ten organoids per slide were randomly selected using ImageJ software, and the mean fluorescence intensity was calculated.

The primary antibodies used for immunofluorescence were rabbit anti‐Ki67 (1:200, CST, 34330SF), rabbit anti‐ZO1 (1:1000, Proteintech, 21773‐1‐AP), rabbit anti‐Occludin (1:1000, Proteintech, 27260‐1‐AP), mouse anti‐E‐cadherin (1:300, CST, 14472A), rabbit anti‐TSC1 (1:50; Beyotime, AF8256), rabbit anti‐NDUFB8 (1:50, HUABIO, ET7108‐25), rabbit anti‐SDHA (1:500, HUABIO, ET1703‐40), rabbit anti‐UQCRFS1 (1:1000, HUABIO, ET7108‐28), and rabbit anti‐MTCO1 (1:250, Abcam, ab203912). The fluorescently labeled secondary antibodies included donkey anti‐rabbit 555 (1:500, Abcam, ab150062), goat anti‐rabbit 488 (1:500, Invitrogen, A‐11008), and goat anti‐mouse 488 (1:500, HUABIO, HA1125).

### 4.27 Scanning Electron Microscope

Organoids were collected in pre‐cooled PBS and incubated at 4°C for 30 min to ensure complete removal of Matrigel. Subsequently, the organoids were washed twice with PBS and resuspended in 2% glutaraldehyde fixative solution at room temperature for 2 h. Dehydration was conducted using a graded ethanol series of 50%, 70%, and 100%. Subsequently, the samples were dried in a supercritical dryer (Quorum K850) for 1 h and coated with gold. Imaging was performed using a scanning electron microscope (HITACHI Regulus 8100).

### 4.28 Transmission Electron Microscopy

Organoids were harvested in ice‐cold PBS and maintained at 4°C for 30 min to ensure thorough dissolution of Matrigel. The organoids were then washed twice with PBS and resuspended in 4% glutaraldehyde fixative. For pre‐embedding, samples were immersed in agarose and fixed in 1% osmium tetroxide in 0.1 M phosphate buffer (pH 7.4) at room temperature for 2 h in the dark. Following graded dehydration at room temperature, the samples were embedded by infiltration with acetone and SPI 812 embedding resin (SPI, 02660‐AB). Ultrathin sections (70 nm) were prepared using an ultramicrotome and mounted on square copper 200‐mesh grids. The Grids were stained with a saturated 2% uranyl acetate ethanol solution and lead citrate to prevent CO_2_ buildup, and then dried overnight in a copper grid box at room temperature. Imaging was conducted using a transmission electron microscope (HITACHI, HT7800).

### 4.29 2D Organoid Growth and Treatment Conditions

Organoids were resuspended in cold PBS and kept at 4°C for 30 min, allowing for full elimination of the Matrigel (Corning, 356231). After two PBS washes, the organoids were plated on the surface of solidified Matrigel. Solidified Matrigel was diluted with PBS at ratios of 0:2 (Hard), 1:2 (Mid), and 2:2 (Soft) to yield a total of 200 µL gel, which was layered into 24‐well plates and incubated at 37°C for 60 min for gelation. Plates were incubated at 37°C for 30 min to allow the organoids to adhere to the surface. Organoid culture medium for colon cancer (bioGenous, K2003‐HC‐500) was added, and 2D growth was monitored under a microscope over five consecutive days. GBOXIN (MedChemExpress, HY‐111651, 30 µM), an OXPHOS inhibitor, was added to the organoid culture medium when required and incubated for 12 h before further analysis. The ADORA2B inhibitor ZM241385 (MedChemExpress, HY‐19532, 50uM) was added to the organoid culture medium when required and incubated for 12 h. Then, the organoids were collected and plated on the surface of solidified Matrigel of different stiffnesses. Quantitative analysis of changes in the organoid area was performed using ImageJ and OrganoID [60].

### 4.30 Immunoblotting

Organoids were collected with ice‐cold PBS, and Matrigel was completely dissolved. After centrifugation, organoids were lysed in RIPA buffer (Biosharp, BL504A) containing protease inhibitor (Biosharp, BL612A) and phosphatase inhibitor Cocktail II (HY‐k0022‐1 mL), incubated on ice for 60 min, and sonicated twice (30 s each, 60 kHz). Lysates were centrifuged at 12 000 rpm for 20 min, and the supernatants were collected and boiled for 8 min. Equal amounts of protein were separated by SDS‐PAGE and transferred onto PVDF membranes (Merck Millipore). After blocking with rapid blocking buffer (Epizyme, PS108) for 15 min, membranes were incubated with primary antibodies at 4°C overnight, followed by incubation with HRP‐conjugated goat anti‐rabbit (Abcam, ab205718, 1:2000) or goat anti‐mouse (Abcam, ab205719, 1:2000) secondary antibodies diluted in 2.5% non‐fat milk in TBST for 1 h at room temperature. Blots were imaged and analyzed using Image Lab software (Bio‐Rad). β‐actin served as the loading control.

### 4.31 Atomic Force Microscopy

LST and PA tissues were collected within 1 h after endoscopic resection, snap‑frozen at ‑80°C, embedded in optimal cutting temperature compound, and cryosectioned into 30 µm sections. Prior to AFM measurement, each section was thawed in DPBS without protease inhibitors (Roche, 04693132001) at room temperature. Quantitative Nanomechanics (QNM) indentation was performed using an MFP‐3D Origin AFM (Asylum Research, Oxford Instruments). Silicon nitride cantilevers with a spring constant of 3 N/m (Bruker, MLCT) and platinum‐coated silicon probes (Multi75E‐G, Budget Sensors) were used. The cantilever was calibrated before each experiment using the thermal oscillation method. Indentation was performed at an approach speed of 0.4 µm/s. Six 20 µm × 20 µm force maps were acquired per sample (3 AFM sections per patient), each consisting of a 5 × 5 raster series of indentations using JPK software. The Hertz model was applied to determine tissue elasticity, assuming a Poisson's ratio of 0.5 for Young's modulus calculation.

### 4.32 Statistical Analysis

All statistical analyses were conducted using R (version 4.2.2) or GraphPad Prism (version 9.0.0), with a two‐sided p‐value of less than 0.05 considered statistically significant. Continuous variables are presented as mean ± standard error of the mean (SEM) for normally distributed data. Categorical variables are expressed as counts (n) and percentages (%). Each experiment was independently repeated at least three times, unless otherwise indicated. The sample size (n) for each statistical analysis or experiment is specified in the corresponding figure legend. Comparisons between two groups were assessed using two‐tailed Student's t‐tests, while comparisons among multiple groups were performed by one‐way ANOVA followed by Tukey's post hoc test. Non‐parametric data were analyzed using the Mann–Whitney U test as appropriate. The time‐course growth data were assessed using two‐way ANOVA followed by Sidak's multiple comparisons test. Categorical variables were compared using the Chi‐square test. For all organoid‐related experiments, all conditions were performed in triplicate and images were acquired on at least 10 organoids per well (n = 3).

Pearson correlation analysis was used to assess the relationships between different cell signatures and gene expression in epithelial cells. To compare the biological characteristics of the cell subtypes, we collected up‐to‐date gene signatures and TME‐related gene lists from prior publications. We visualized the differential expression of these target variables across cell clusters using ComplexHeatmap, pheatmap, and scRNAtoolVis packages.

The specific statistical tests used are detailed in the Results section and figure legends with significance levels indicated as follows: *p < 0.05, **p < 0.01, ***p < 0.001, ****p < 0.0001, ns: not significant.

## Ethics Approval

This study was approved by the Medical Ethics Committee of Nanfang Hospital, Southern Medical University (registration number: NFEC‐2024‐466). All participants provided written informed consent prior to study participation.

## Conflicts of Interest

The authors declare no competing interests.

## Supporting information




**Supporting File 1**: advs74825‐sup‐0001‐SuppMat.docx.

**Supporting File 2**: advs74825‐sup‐0002‐FigureS1‐S8.zip.

**Supporting File 3**: advs74825‐sup‐0003‐TableS1‐S5.zip.

## Data Availability

Sequencing data have been deposited in the Genome Sequence Archive at the National Genomics Data Center, China National Center for Bioinformation / Beijing Institute of Genomics, Chinese Academy of Sciences (available at: https://ngdc.cncb.ac.cn), under accession numbers HRA009002 and HRA008719. All data generated or analyzed in this study are included in the online supplemental methods. The data, analytical methods, and research materials in this paper will be available from the corresponding authors upon reasonable request.
