# Supplementary material for: A Soft Matrix Microenvironment Promotes Laterally Spreading Tumors via Oxidative Phosphorylation‐Dependent Cell Adhesion
Source: Adv Sci (Weinh). 2026 Mar 15;13(30):e23872. doi: 10.1002/advs.202523872 (PMC13248847; doi:10.1002/advs.202523872)
Supplement: Supplementary file 1 — Supporting File 1: advs74825‐sup‐0001‐SuppMat.docx. [file ADVS-13-e23872-s001.docx]

Supporting Information

**A Soft Matrix Microenvironment Promotes Laterally Spreading Tumors via Oxidative Phosphorylation-Dependent Cell Adhesion**

*Jiamin Zhong, Jingyi Lu, Haopeng Li, Jun Zhong, Xiaobei Luo, Yiyi Hu, Xianfei Wang, Pengfei Wang, Yanning Zhang, Zhenjiang Wang, Qiuhua Lai, Zhenyu Chen, Wenting Mi, Wang Tin San-to, Weize Li, Shuping Tan, Qihong Cheng, Ruijia Li, Yida Nie, Side Liu,^*^ Bing Huang,^*^ Zelong Han^*^*

**This file includes:**

Figure S1 to S8

Captions for Table S1 to S5

**Other Supplementary Materials for this manuscript include the following:**

Table S1 to S5


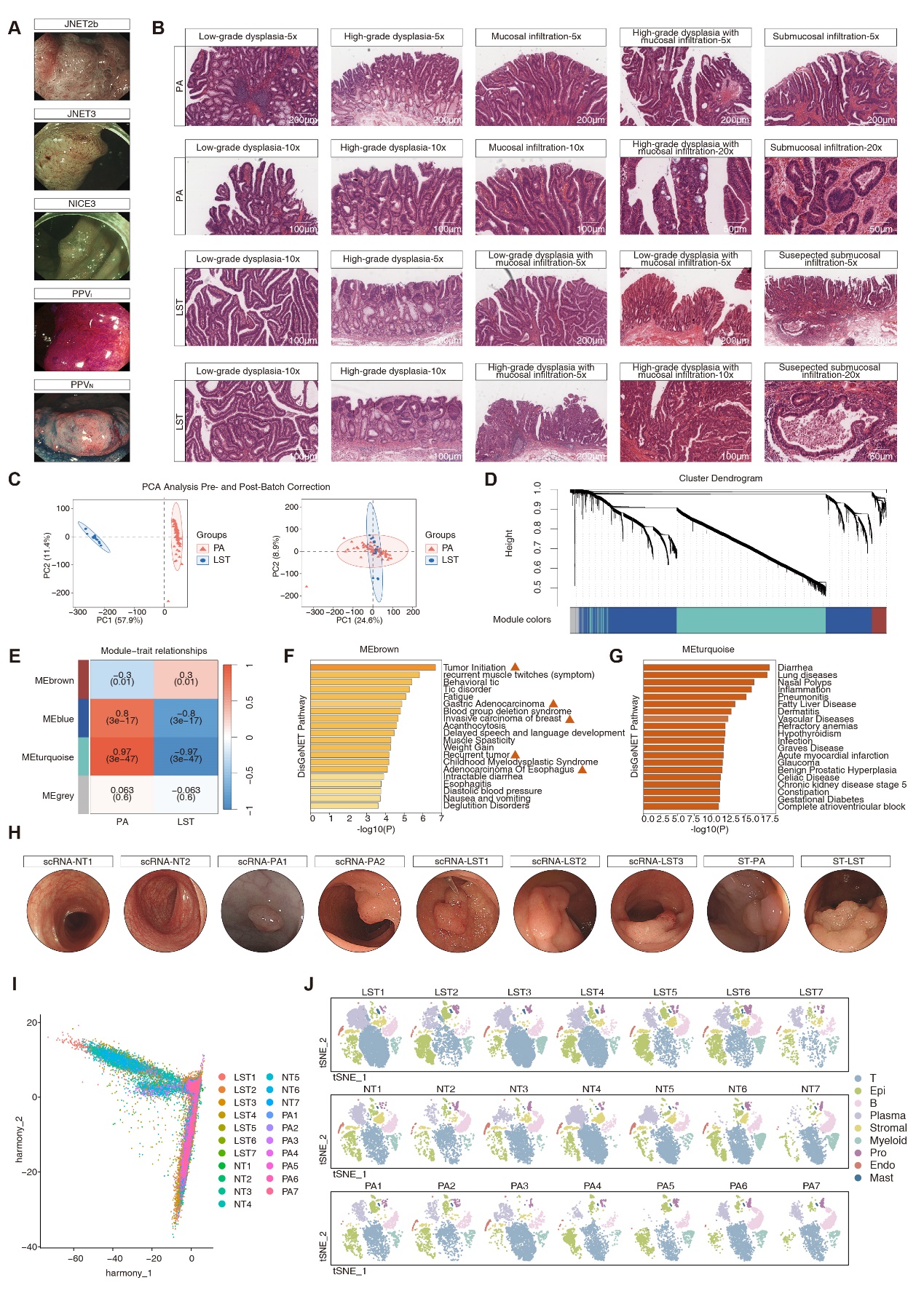


**Figure S1. Malignant potential of laterally spreading tumors**

*(A)* Representative endoscopic images of distinct endoscopic subtypes of LSTs.

*(B)* Representative histopathological images from PA and LST tissues, showcasing classifications such as low-grade dysplasia, high-grade dysplasia, mucosal invasion with low-grade dysplasia, mucosal invasion with high-grade dysplasia, and submucosal infiltration.

*(C)* Principal Component Analysis (PCA) plots for two datasets before (left) and after (right) batch effect removal.

*(D)* Dendrogram illustrating gene clustering into modules via hierarchical clustering, with colors representing distinct modules.

*(E)* Module-trait heatmap showing the correlation between PA and LST traits and identified module eigengenes.

*(F)* Enrichment analysis of eigengenes from module MEbrown.

*(G)* Enrichment analysis of eigengenes from module MEturquoise.

*(H)* Representative endoscopic images of sequenced samples.

*(I)* Principal Component (PC) plot post-Harmony integration, colored by patient origin.

*(J)* t-SNE visualization showing the distribution of major cell types across all patients.


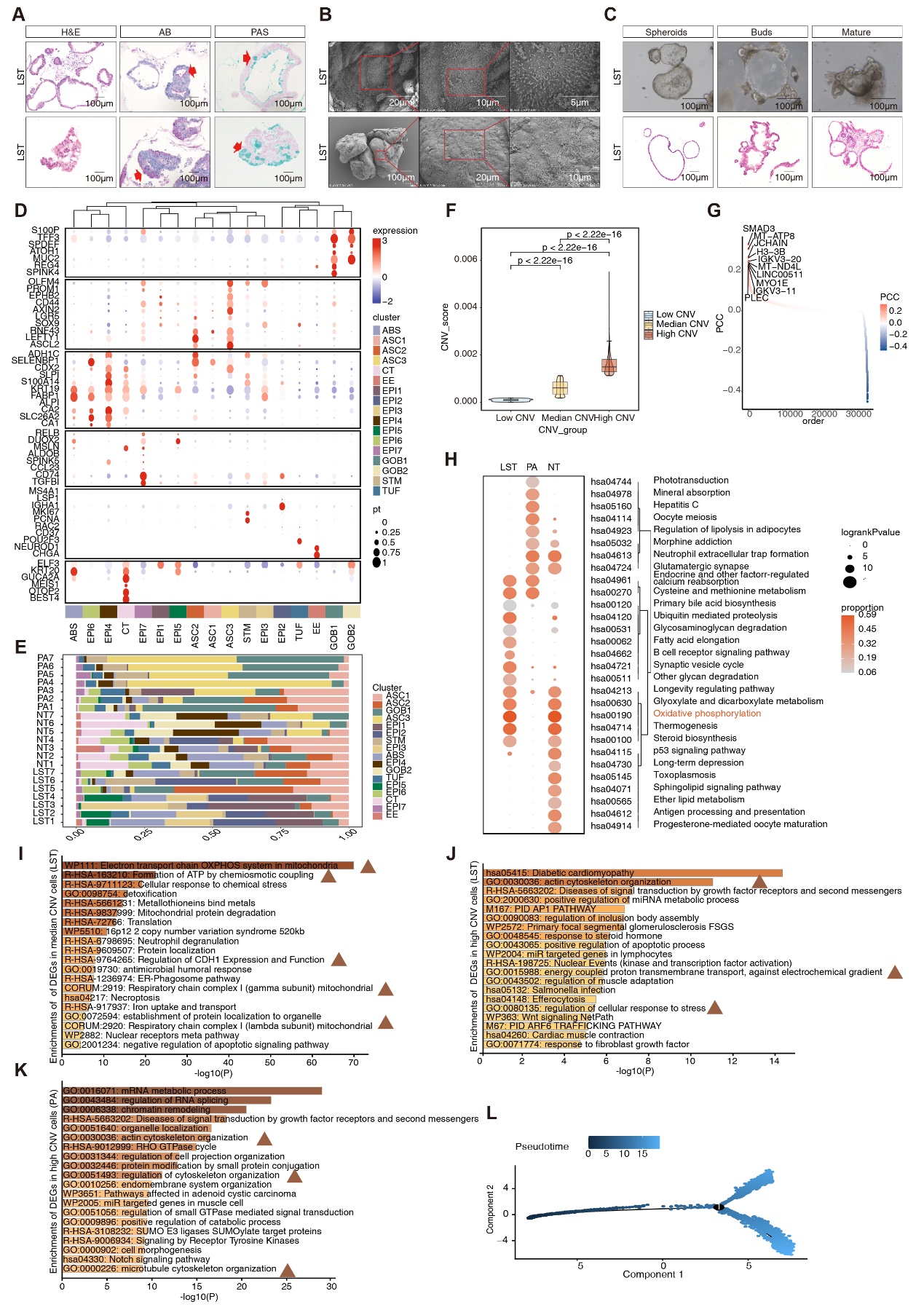


**Figure S2. Co-expression network construction of epithelial cells**

*(A)* Representative H&E and PAS-AB staining images highlighting goblet cells (indicated by red arrows) (n=3).

*(B)* Representative scanning electron microscopy images of organoids derived from LSTs, showing intricate internal architecture with fine structural details (upper panels) and comprehensive three-dimensional morphology with external surface characteristics (lower panels) (n=3).

*(C)* Representative bright-field and H&E images (40× magnification) of different organoid stages (spheroids, buds, and mature types) (n=3) from LSTs.

*(D)* Dot plot showing marker genes associated with epithelial subtypes.

*(E)* Histograms illustrating the distribution of the epithelial subtypes across all samples.

*(F)* Box plot showing the CNV levels of each CNV-defined group.

*(G)* Scatter plot illustrating heritability-correlated genes identified by scPagwas.

*(H)* Dot plot showing specific genetics pathway for each group identified by scPagwas.

*(I)* Bar plot illustrating the enrichment of marker genes from inferred median-CNV cells in LSTs, as analyzed by Metascape.

*(J)* Bar plot illustrating the enrichment of marker genes from inferred high-CNV cells in LSTs, as analyzed by Metascape.

*(K)* Bar plot illustrating the enrichment of marker genes from inferred high-CNV cells in PAs, as analyzed by Metascape.

*(L)* Pseudotime trajectory of cells along the direction of developmental progression.


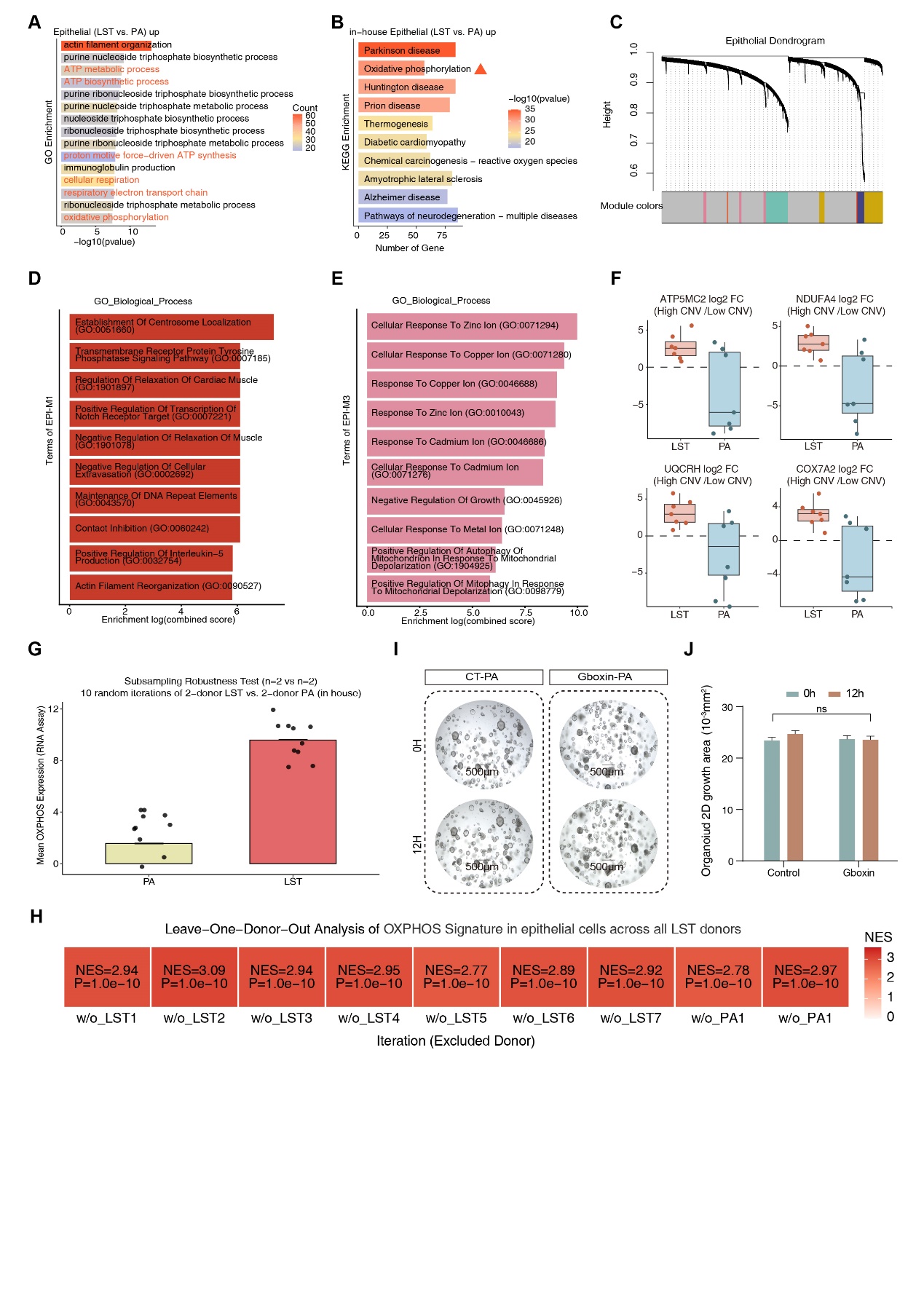


**Figure S3. Upregulation of oxidative phosphorylation as a key event in LST progression**

1. GO analysis showing pathways upregulated in epithelial cells from LSTs compared with PAs.

*(B)* KEGG enrichment analysis restricted to the in-house cohort confirms the

significant upregulation of OXPHOS in LST versus PA epithelial cells.

*(C)* The hdWGCNA dendrogram showing the different co-expression modules resulting from the network analysis. Each color indicates the co-expression module assignment.

*(D)* GO enrichment analysis of genes in Epi-Module 1.

*(E)* GO enrichment analysis of genes in Epi-Module 3.

*(F)* Box plots comparing the distribution of log2 FC (CNV-high vs. CNV-low) for the OXPHOS-related genes between LST and PA groups. Each dot represents an individual patient. The dashed line indicates y = 0 (no difference).

*(G)* Bar plot showing the mean expression of the OXPHOS signature across 10 independent stochastic iterations (n=2 PA vs. n=2 LST donors).

*(H)* Heatmap illustrating the robust and consistently positive enrichment of the OXPHOS signature in LST cells relative to PA cells across all leave-one-donor-out permutations. The heatmap presents the Normalized Enrichment Scores (NES) and adjusted p-values (P_adj) for key biological signatures across nine independent iterations. In each iteration, a single donor was sequentially excluded (denoted as “w/o”, e.g., “w/o_LST1” indicates analysis performed after excluding donor LST1).

*(I)* Representative microscope bright-field images (4× magnification) of PA organoids treated with the OXPHOS inhibitor Gboxin at 0 h and 12 h.

*(J)* Bar plot depicting statistics analysis of changes in the organoid area from (E) (n=3 for each group).

All P values were calculated using the Mann-Whitney U test. *p < 0.05, **p < 0.01, ***p < 0.001, ****p < 0.0001.


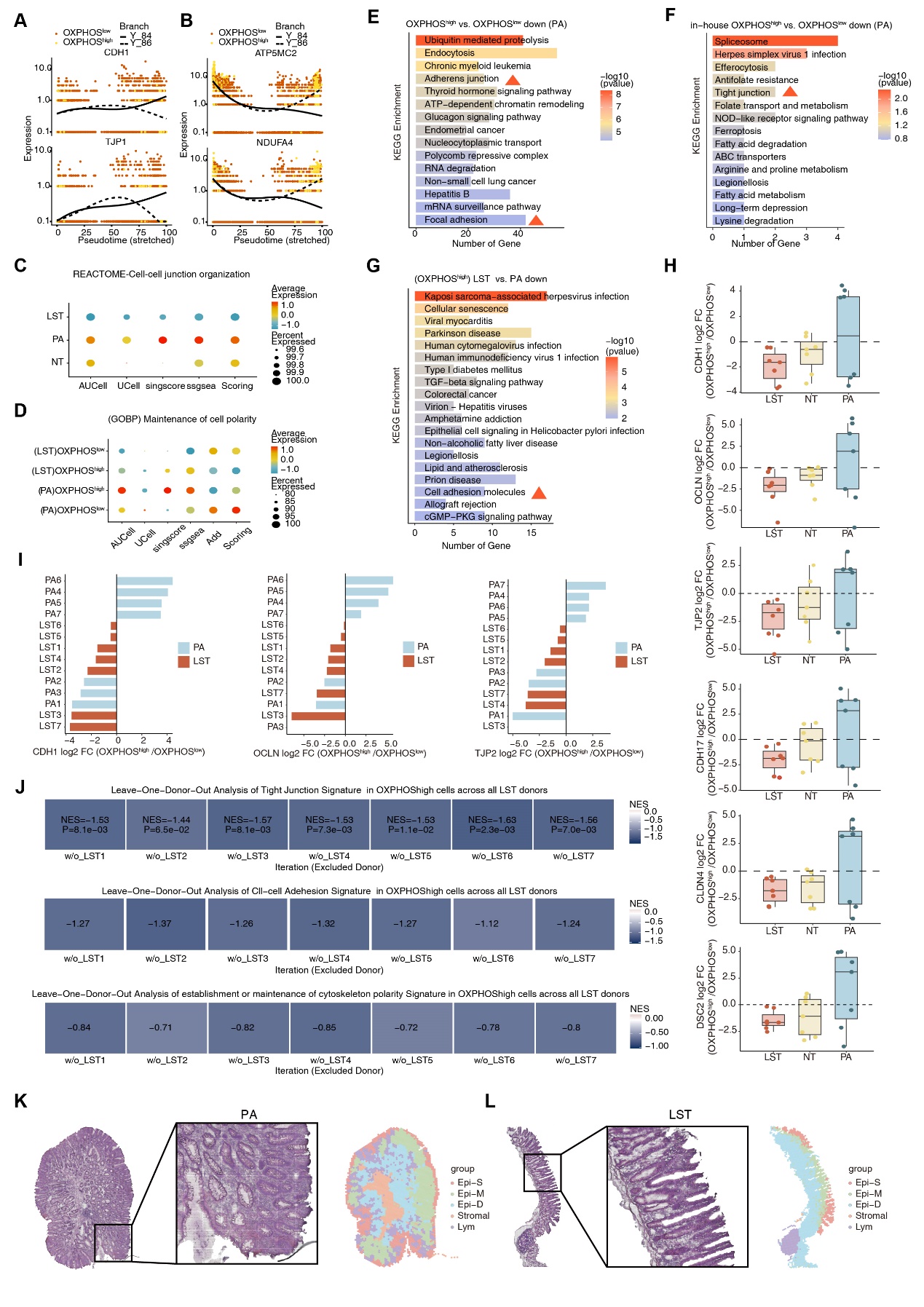


**Figure S4. Increased oxidative phosphorylation drives the downregulation of adhesion molecules**

*(A-B)* Pseudotime trajectory analysis of cell-junction molecules CDH1 and TJP1 (left) and OXPHOS-related molecules ATP5MC2 and NDUFA4 (right). Gene expression dynamics are shown with pseudotime on the x-axis and expression levels on the y-axis, with colors indicating different OXPHOS differentiation states. Solid and dashed lines represent the two post-branch differentiation trajectories, respectively.

*(C)* Dot plot illustrating the signature scores of cell-cell junction organization across different groups.

*(D)* Dot plot illustrating the signature scores of maintenance of cell polarity across different groups.

(*E*) KEGG enrichment analysis of DEGs downregulated in OXPHOS^high^ cells relative to OXPHOS^low^ cells within PA samples

*(F)* KEGG enrichment analysis of DEGs downregulated in OXPHOS^high^ cells relative to OXPHOS^low^ cells within in-house PA samples.

(*G*) KEGG enrichment analysis of DEGs downregulated in OXPHOS^high^ LST cells compared with OXPHOS^low^ PA cells.

(*H*) Bar plots showing the log2 fold change (FC) of key adhesion molecules between OXPHOS^high^ and OXPHOS^low^ cells in individual samples from LSTs and PAs. Positive and negative values indicate higher expression in OXPHOS^high^ and OXPHOS^low^ populations, respectively.

(*I*) Box plots comparing the distribution of log2 FC (OXPHOS^high^ vs. OXPHOS^low^) for the indicated genes across LST, NT, and PA groups. Each dot represents an individual patient. The dashed line indicates y = 0 (no difference).

*(J)* Heatmap illustrating the robust and consistently positive enrichment of the Tight junction, Cell-cell adhesion and Cytoskeleton polarity signatures in LST cells relative to PA cells across all leave-one-donor-out permutations. The heatmap presents the Normalized Enrichment Scores (NES) and adjusted p-values (P_adj) for key biological signatures across nine independent iterations. In each iteration, a single donor was sequentially excluded (denoted as “w/o”, e.g., “w/o_LST1” indicates analysis performed after excluding donor LST1).

*(K-L)* H&E staining images and spatial clustering analysis based on spatial transcriptome spots: *(K)* PAs and *(L)* LSTs.


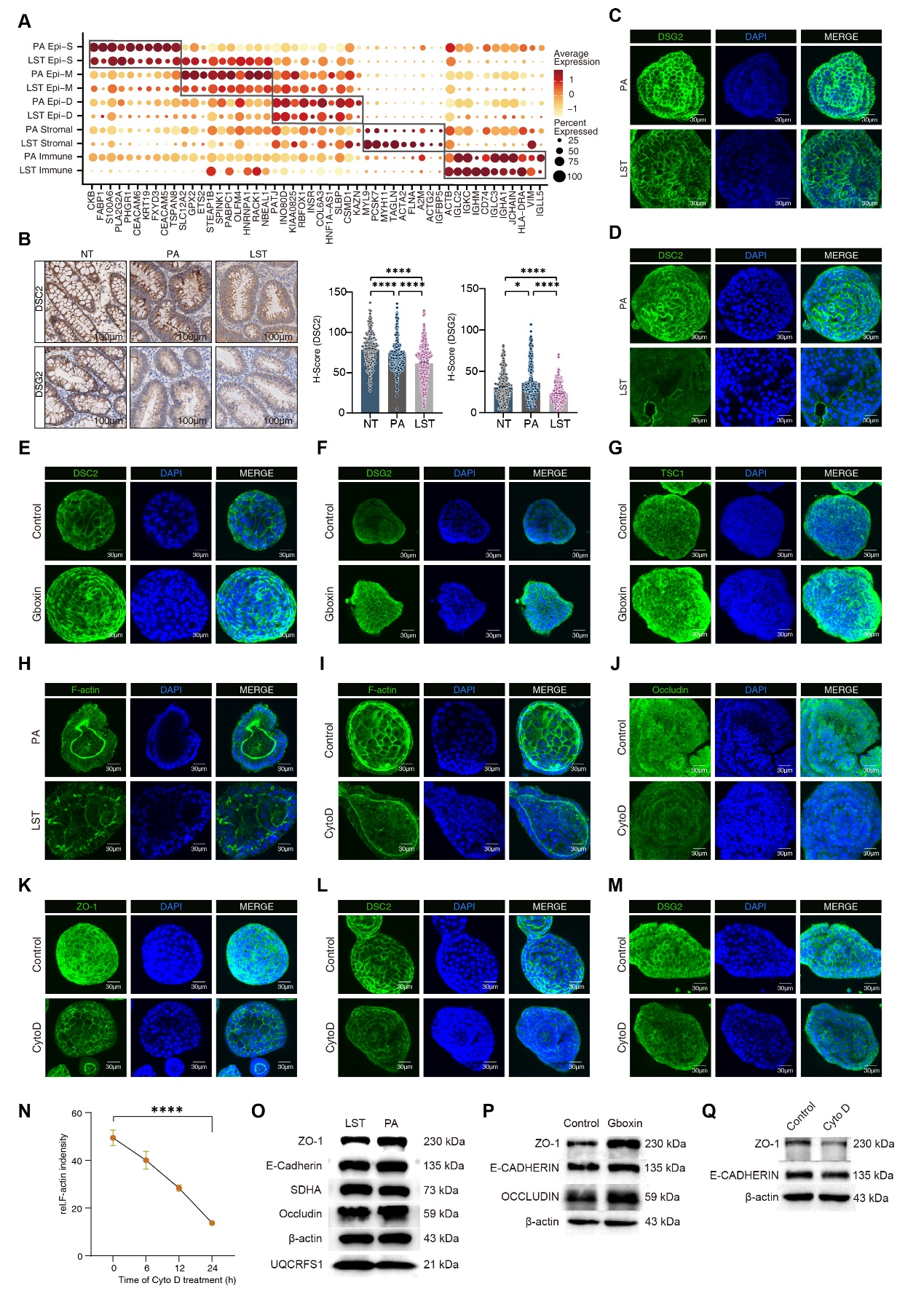


**Figure S5. Elevated OXPHOS levels drive the downregulation of adhesion molecules by promoting cytoskeleton depolymerization during LST progression**

*(A)* Dot plot displaying marker expression levels across distinct spatial regions in both groups.

*(B)* Representative IHC images of cell junction components (DSC2 and DSG2) across three groups (left), with corresponding quantification shown in bar plots (right) (n = 15 per group). P values were calculated using the one-way ANOVA test. *p < 0.05, **p < 0.01, ***p < 0.001, ****p < 0.0001.

*(C)* Representative immunofluorescence images of DSG2 in LST and PA organoids (n=3 for each group).

*(D)* Representative immunofluorescence images of DSC2 in LST and PA organoids (n=3 for each group).

*(E)* Immunofluorescence analysis of DSC2 with treatment of Gboxin in LST organoids (n=3 for each group).

*(F)* Immunofluorescence analysis of DSG2 with the treatment of Gboxin for 12 hours in LST organoids (n=3 for each group).

*(G)* Immunofluorescence analysis of TSC1 with the treatment of Gboxin in LST organoids (n=3 for each group).

*(H)* Immunofluorescence analysis of cytoskeleton marker F-actin in PA and LST organoids (n=3 for each group).

*(I)* Immunofluorescence analysis of cytoskeleton marker F-actin with treatment of cytochalasin D (100nM) for 12 hours in LST organoids (n=3 for each group).

*(J)* Immunofluorescence analysis of cell junction molecules Occludin with the treatment of cytochalasin D (100nM) for 12 hours in LST organoids (n=3 for each group).

*(K)* Immunofluorescence analysis of cell junction molecules ZO-1 with the treatment of cytochalasin D (100nM) for 12 hours in LST organoids (n=3 for each group).

*(L)* Immunofluorescence analysis of cell junction molecules DSC2 with the treatment of cytochalasin D (100nM) for 12 hours in LST organoids (n=3 for each group).

*(M)* Immunofluorescence analysis of cell junction molecules DSG2 with the treatment of cytochalasin D (100nM) for 12 hours in LST organoids (n=3 for each group).

*(N)* Immunofluorescence analysis of F-actin in LST organoids treated with 100 nM Cytochalasin D across a time gradient (0, 6, 12, and 24 h). Data are presented as mean ± SEM (n=3). P values were calculated using the one-way ANOVA test. *p < 0.05, **p < 0.01, ***p < 0.001, ****p < 0.0001.

*(O)* Tight junction (ZO-1, E-Cadherin and Occludin) and OXPHOS (SDHA, UQCRFS1) expression in LST and PA organoids, were assayed by Western blotting (n = 3).

*(P)* Tight junction (ZO-1, E-Cadherin and Occludin) expression in LST organoids following Gboxin inhibition, were assayed by Western blotting (n = 3).

*(Q)* Tight junction (ZO-1 and E-Cadherin) expression in LST organoids following Cytochalasin D inhibition, were assayed by Western blotting (n = 3).


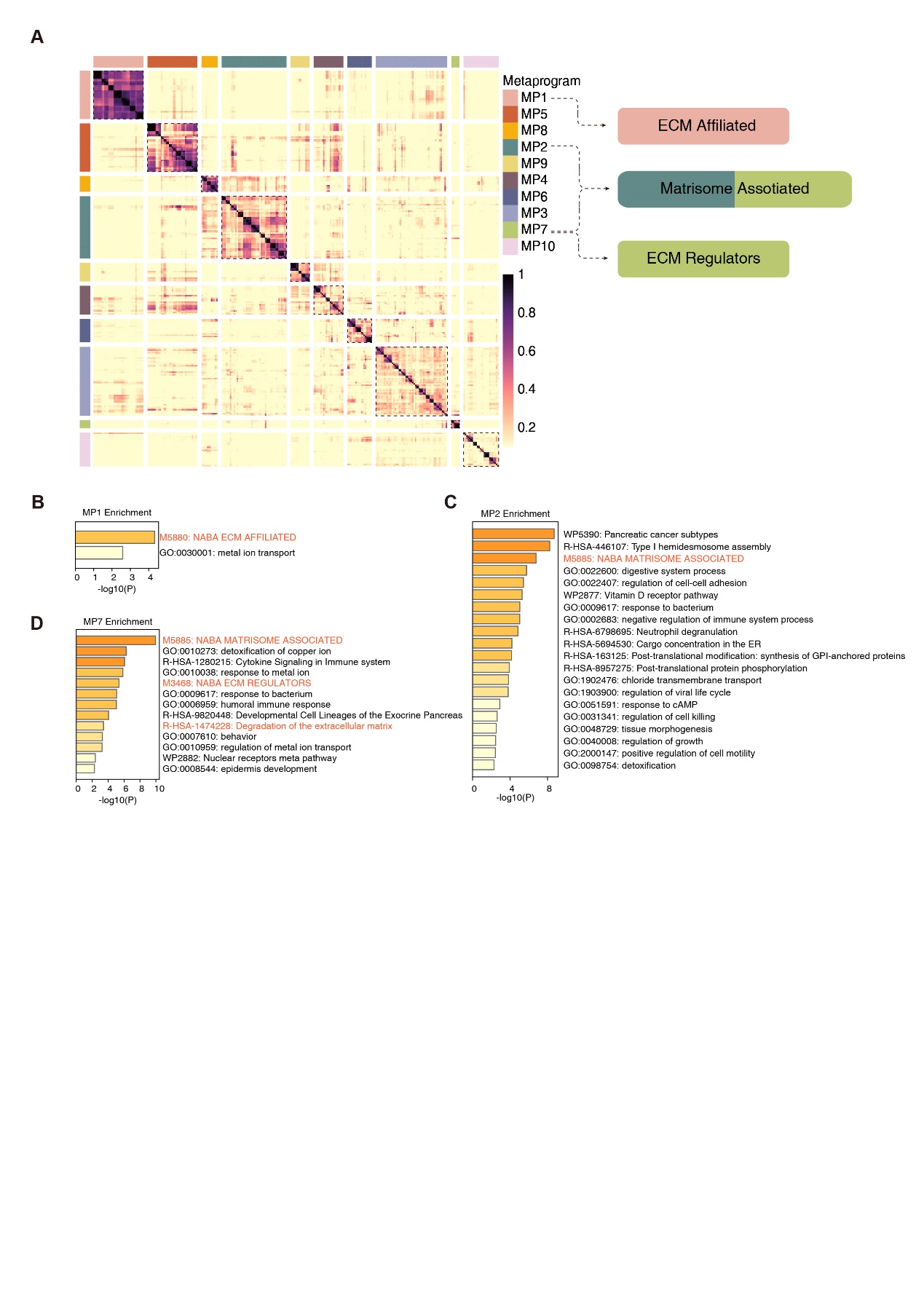


**Figure S6. Identification of coherent gene modules underlying transcriptional programs in LST epithelium**

*(A)* Heatmap showing the pairwise similarity of metaprograms (MPs) derived from LST patients.

Rows and columns represent consistent NMF programs across LST samples, and the color of each block indicates the correlation coefficient between two programs.

*(B)* Bar plot illustrating the enrichment of MP1 genes as analyzed by Metascape.

*(C)* Bar plot illustrating the enrichment of MP2 genes as analyzed by Metascape.

*(D)* Bar plot illustrating the enrichment of MP7 genes as analyzed by Metascape.


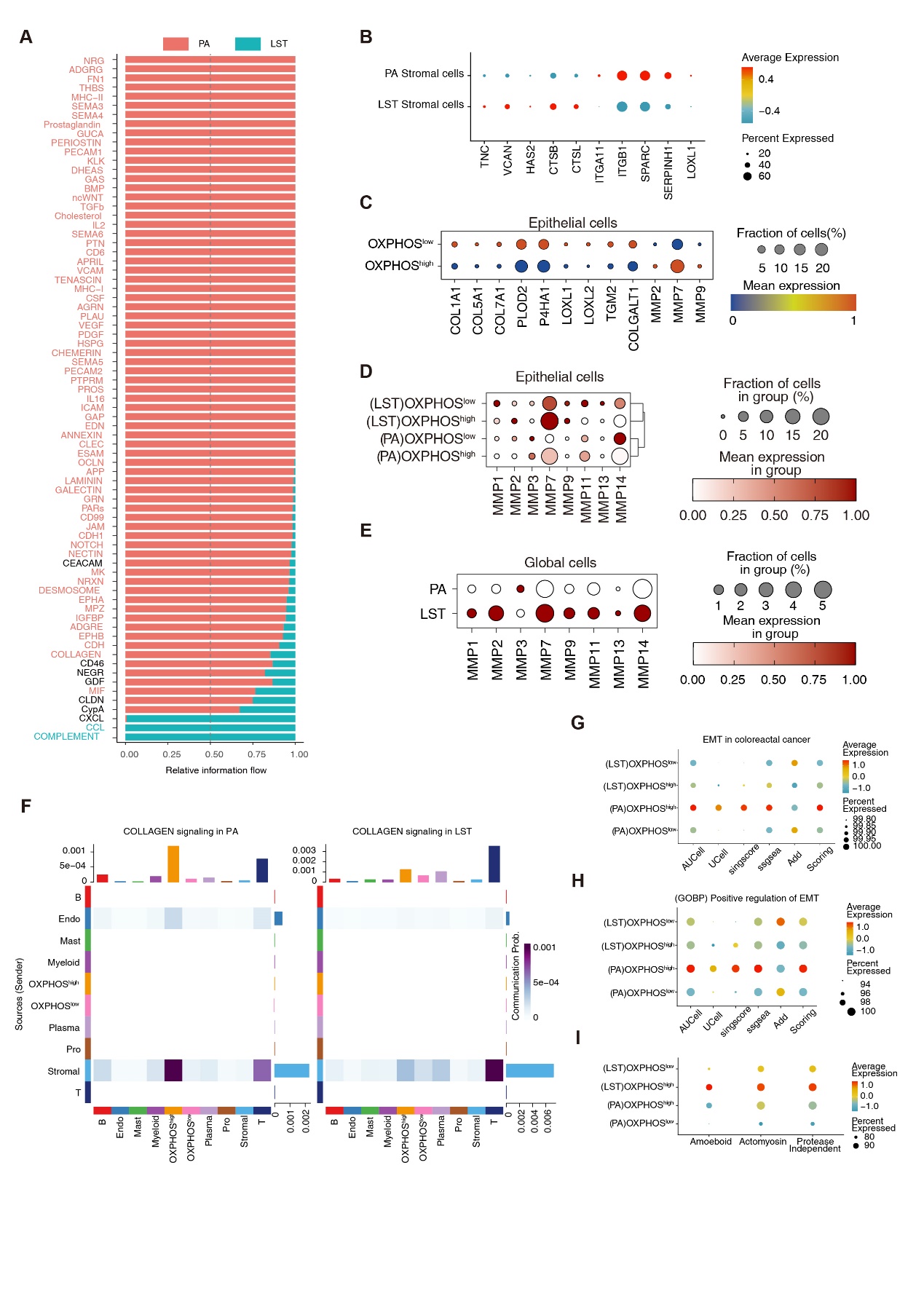


**Figure S7. Stromal cells shape the developmental patterns of PAs and LSTs by modulating ECM dynamics**

*(A)* The bar chart showing the comparison of the overall information flow of each signaling pathway. The top signaling pathways colored red are enriched in PA, and those colored greens are enriched in LSTs.

*(B)* Dot plot comparing the expression of key stromal markers and matrix-stabilizing genes (e.g., SPARC, SERPINH1, LOXL1) between PA and LST stromal cells. The color scale reflects average expression, while dot size indicates the percentage of cells expressing each marker.

*(C)* Dot plot illustrating the expression of collagen family genes (e.g., COL1A1, COL5A1) and matrix-modifying enzymes (e.g., P4HA1, LOXL1) in OXPHOS^high^ versus OXPHOS^low^ epithelial cells. The dot size represents the fraction of cells expressing the gene, and the color gradient indicates the mean expression level.

*(D)* Dot plot showing the expression profiles of MMP family members across OXPHOS^high^ and OXPHOS^low^ subpopulations within LST and PA epithelial cells. Dendrogram on the right represents the hierarchical clustering of the four groups based on their MMP expression signatures.

*(E)* Comparative dot plot of MMP family gene expression in global cell populations of LST versus PA lesions. This panel highlights the overall enrichment of proteolytic enzymes (e.g., MMP1, MMP7, MMP14) in the LST microenvironment compared to PA.

(*F*) Comparative CellChat analysis of collagen signaling. Heatmaps illustrate communication probabilities between cell types, with stromal cells acting as the predominant ligand senders in both PA and LST groups.

(*G*) Dot plot illustrating the gene set enrichment scores for 'EMT in colorectal cancer' across OXPHOS^high^ and OXPHOS^low^ subpopulations within LST and PA epithelial cells. Scores were calculated using five independent algorithms (AUCell, UCell, singscore, ssGSEA, and AddModuleScore), with the 'Scoring' column representing the integrated sum of these five methodologies.

(*H*) Dot plot illustrating the gene set enrichment scores for 'positive regulation of EMT' across OXPHOS^high^ and OXPHOS^low^ subpopulations within LST and PA epithelial cells. Scores were calculated using five independent algorithms (AUCell, UCell, singscore, ssGSEA, and AddModuleScore), with the 'Scoring' column representing the integrated sum of these five methodologies.

(*I*) Dot plot illustrating the gene set enrichment scores for 'amoeboid', 'Actomyosin', and 'protease independent process' across OXPHOS^high^ and OXPHOS^low^ subpopulations within LST and PA epithelial cells. Scores were calculated using AddModuleScore.


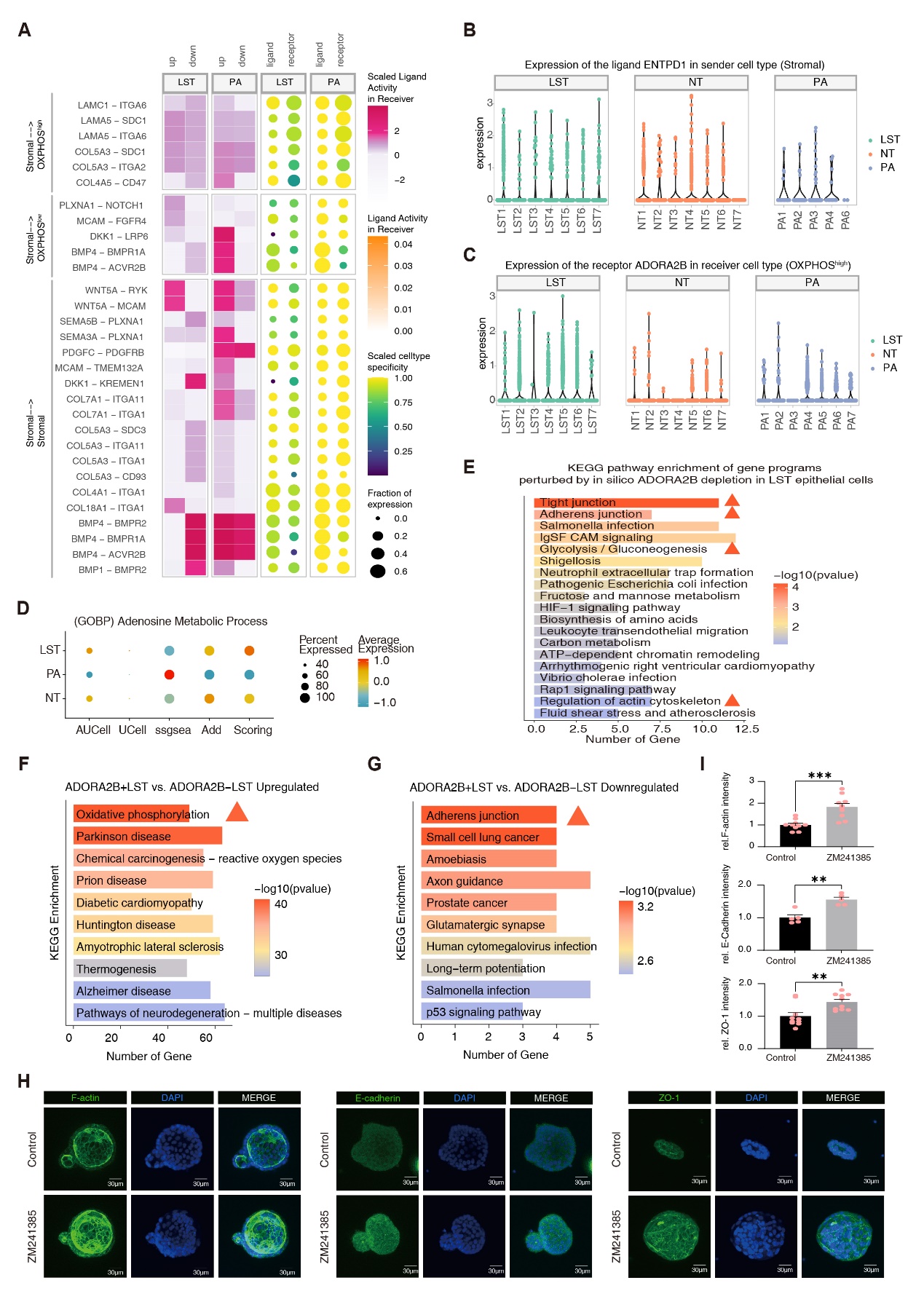


**Figure S8. Intercellular communication analysis revealed the active ligand–receptor pair ENTPD1–ADORA2B in LSTs**

*(A)* Bubble plots depicting the top interactions between OXPHOS^high^, OXPHOS^low^, and stromal cells in PAs, with stromal cells designated as the senders.

*(B)* Violin plot showing the expression levels of the ligand ENTPD1 in stromal cells across samples from the three groups.

*(C)* Violin plot illustrating the expression levels of the receptor ADORA2B in OXPHOS^high^ cells across samples from the three groups.

*(D)* Dot plot illustrating the gene set enrichment scores for 'Adenosine metabolic process' (GOBP) across LST, PA, and NT epithelial groups. Scores were calculated using four independent algorithms (AUCell, UCell, ssGSEA, and AddModuleScore), with the 'Scoring' column representing the integrated sum of these four methodologies.

*(E)* Bar plot showing the KEGG pathway enrichment analysis of the top 200 genes perturbed following in silico ADORA2B depletion in LST epithelial cells.

*(F)*KEGG pathway enrichment analysis of genes upregulated in ADORA2B+LST epithelial cells compared to the ADORA2B-LST subpopulation.

*(G)*KEGG pathway enrichment analysis of genes downregulated in ADORA2B+LST epithelial cells compared to the ADORA2B-LST subpopulation.

*(H)* Immunofluorescence analysis of junctional and cytoskeletal proteins in LST organoids following treatment with the ADORA2B inhibitor (ZM241385).

*(I)* Bar plot showing relative intensities of the immunofluorescence signals (n = 3 for each group).

**Table S1.**

Clinical characterization for all cohorts, related to Figure 1

**Table S2.**

Differentially expressed genes of scRNA cellular subsets

**Table S2(A).**

DEGs of major cell types, related to Figure 1

**Table S2(B).**

DEGs of epithelial cell types, related to Figure 2

**Table S3.**

Differentially expressed genes of ST data regions

**Table S3(A).**

DEGs of 5 regions of PA spatial transcriptomics, related to Figure 3

**Table S3(B).**

DEGs of 5 regions of LST spatial transcriptomics, related to Figure 3

**Table S4.**

Genes of KEGG_OXIDATIVE_PHOSPHORYLATION used to identify OXPHOShigh and OXPHOSlow cells, related to Figure 4

**Table S5.**

Univariate and multivariate analysis of factors affecting submucosa
