# Supplementary figures and images for: A Soft Matrix Microenvironment Promotes Laterally Spreading Tumors via Oxidative Phosphorylation‐Dependent Cell Adhesion
Source: Adv Sci (Weinh). 2026 Mar 15;13(30):e23872. doi: 10.1002/advs.202523872 (PMC13248847; doi:10.1002/advs.202523872)

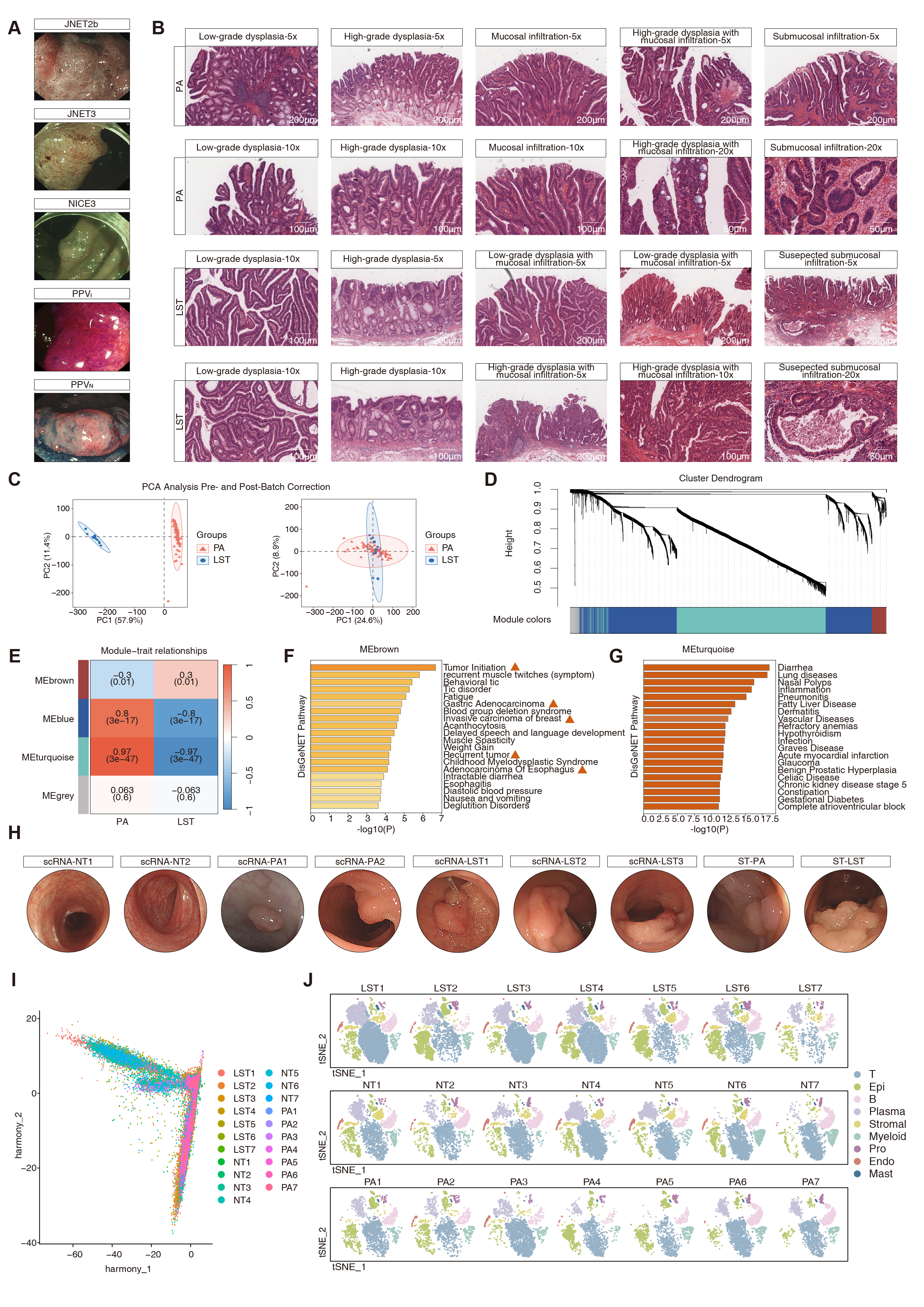

Supplement: Supplementary file 2 — Supporting File 2: advs74825‐sup‐0002‐FigureS1‐S8.zip. [file ADVS-13-e23872-s002.zip › sFig-1.jpg]

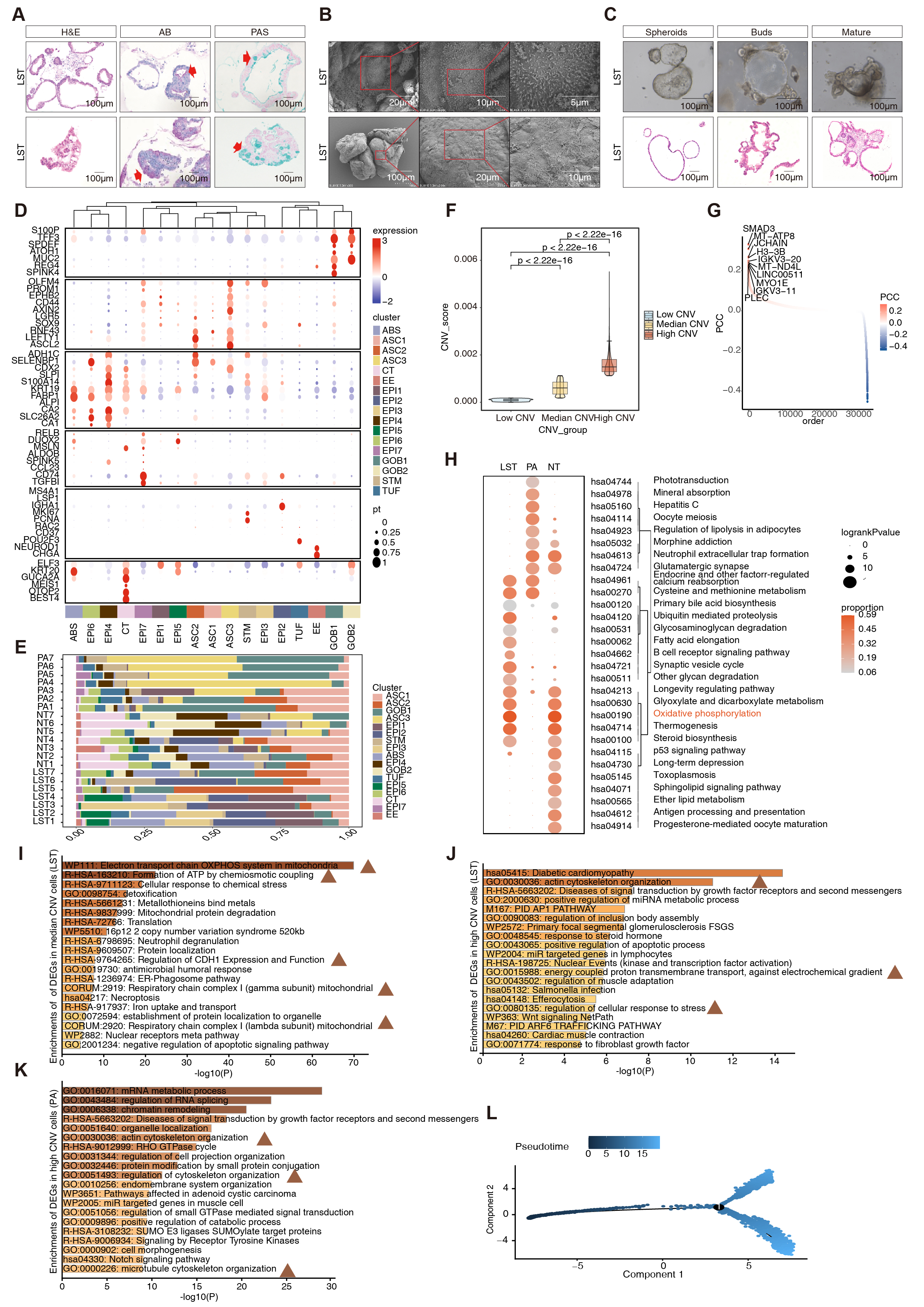

Supplement: Supplementary file 2 — Supporting File 2: advs74825‐sup‐0002‐FigureS1‐S8.zip. [file ADVS-13-e23872-s002.zip › sFig-2.jpg]

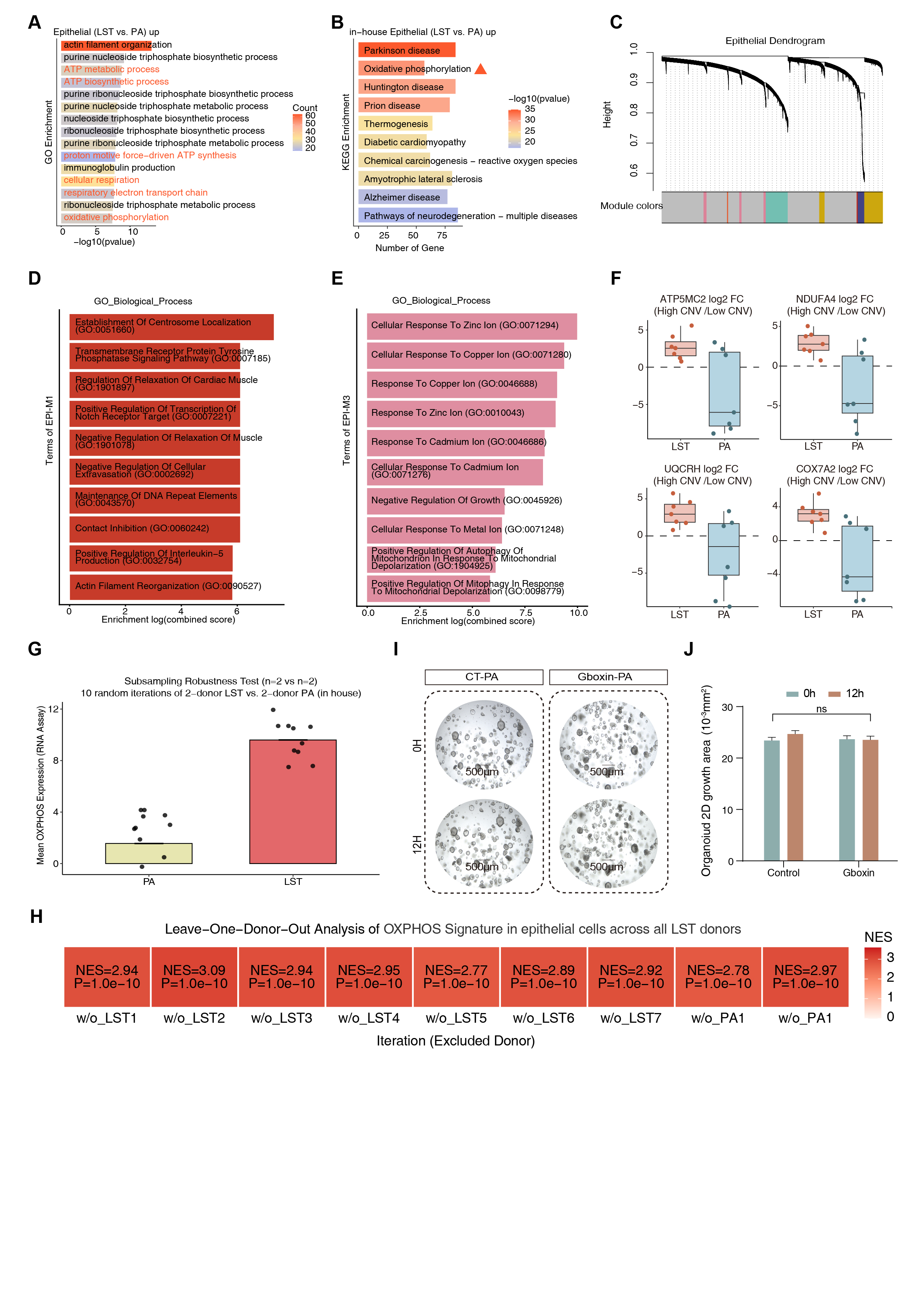

Supplement: Supplementary file 2 — Supporting File 2: advs74825‐sup‐0002‐FigureS1‐S8.zip. [file ADVS-13-e23872-s002.zip › sFig-3.jpg]

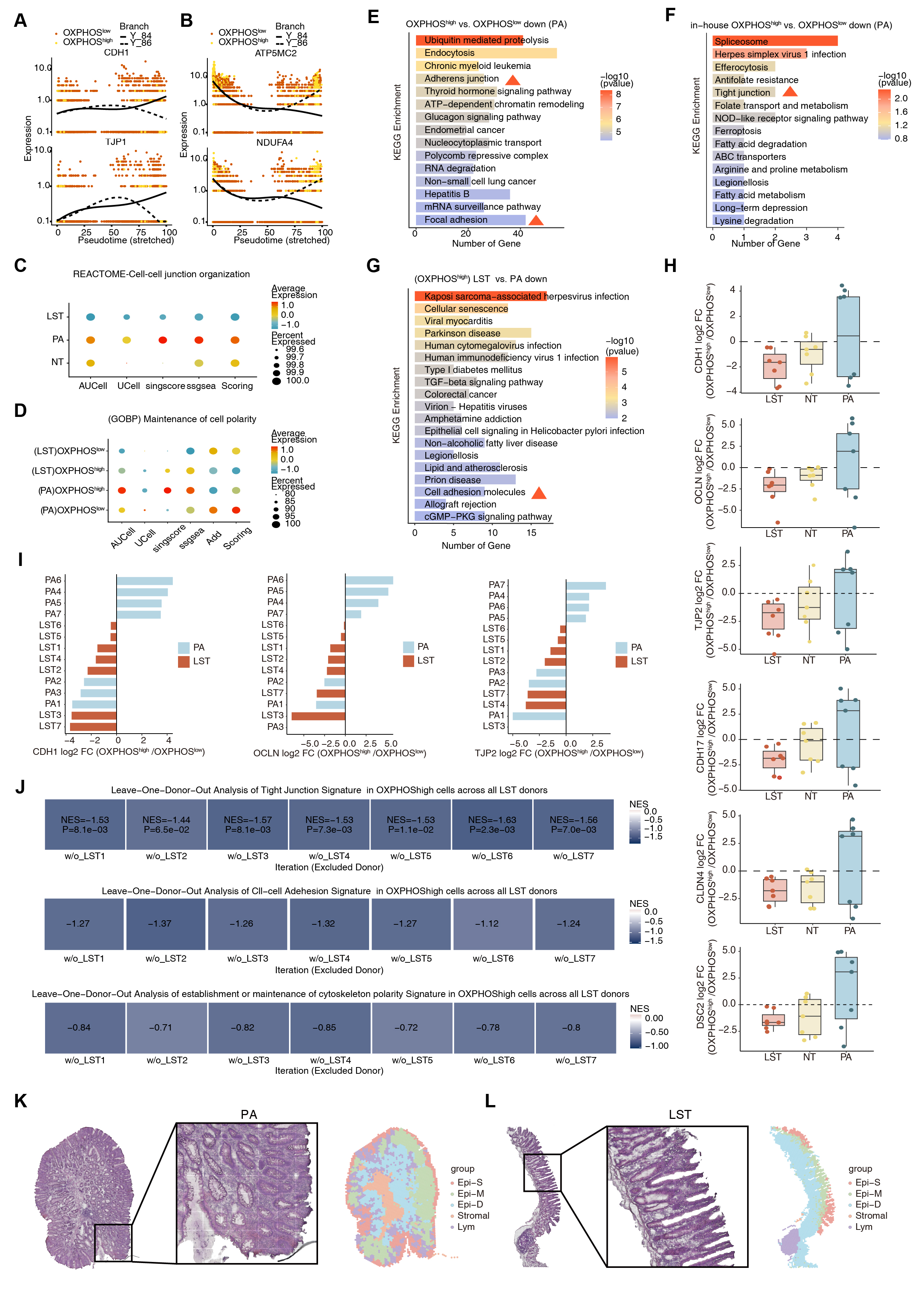

Supplement: Supplementary file 2 — Supporting File 2: advs74825‐sup‐0002‐FigureS1‐S8.zip. [file ADVS-13-e23872-s002.zip › sFig-4.jpg]

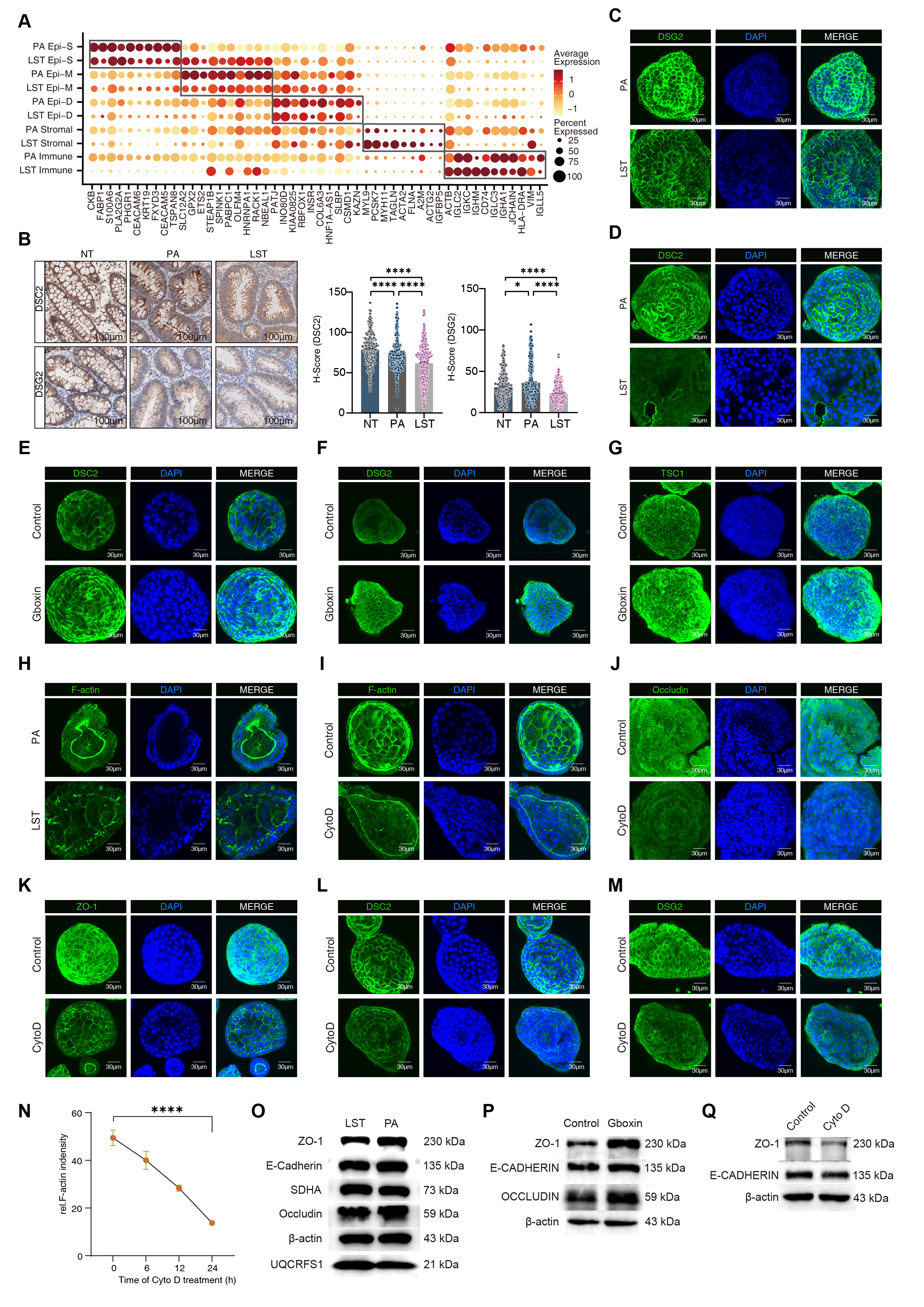

Supplement: Supplementary file 2 — Supporting File 2: advs74825‐sup‐0002‐FigureS1‐S8.zip. [file ADVS-13-e23872-s002.zip › sFig-5.jpg]

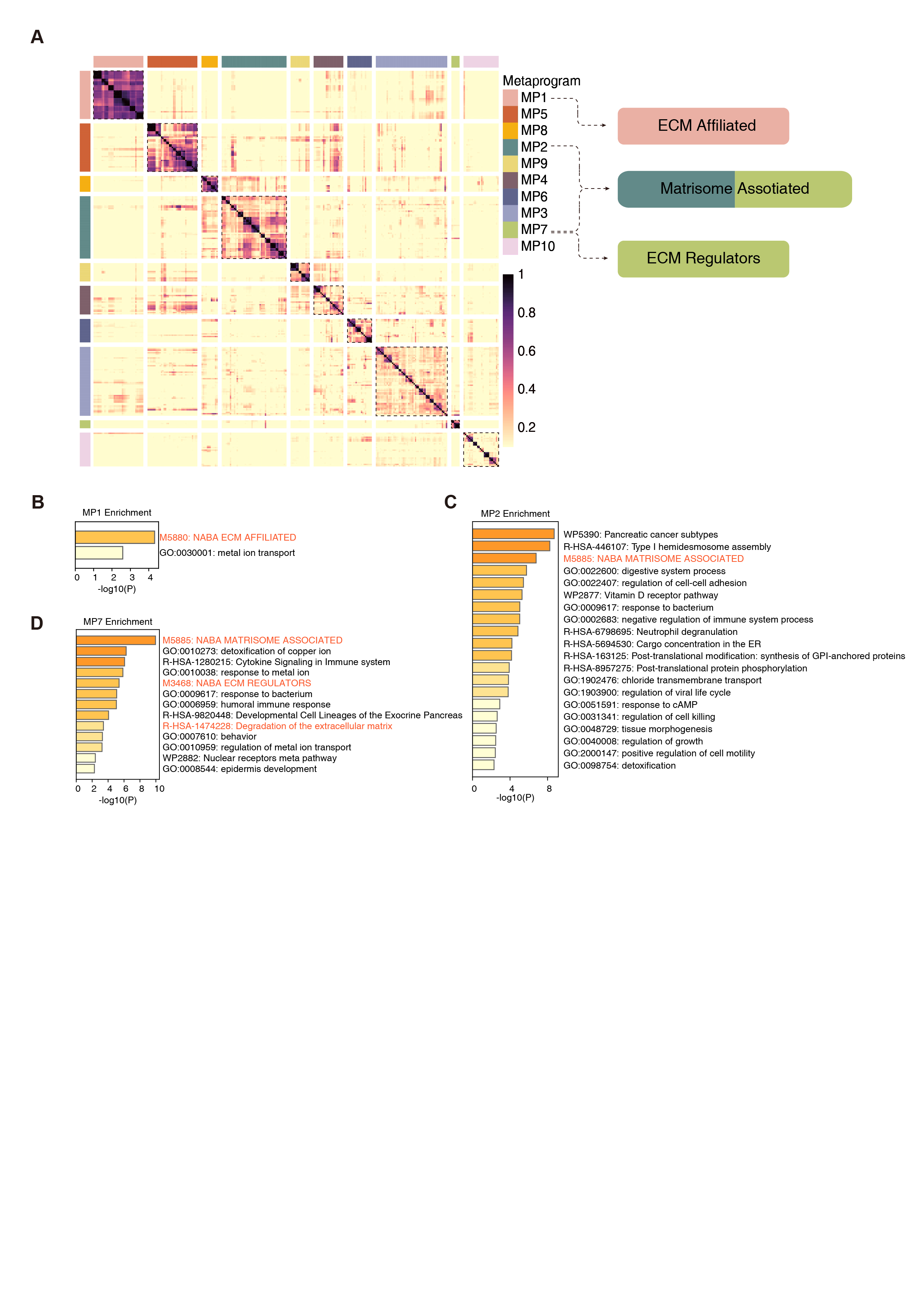

Supplement: Supplementary file 2 — Supporting File 2: advs74825‐sup‐0002‐FigureS1‐S8.zip. [file ADVS-13-e23872-s002.zip › sFig-6.jpg]

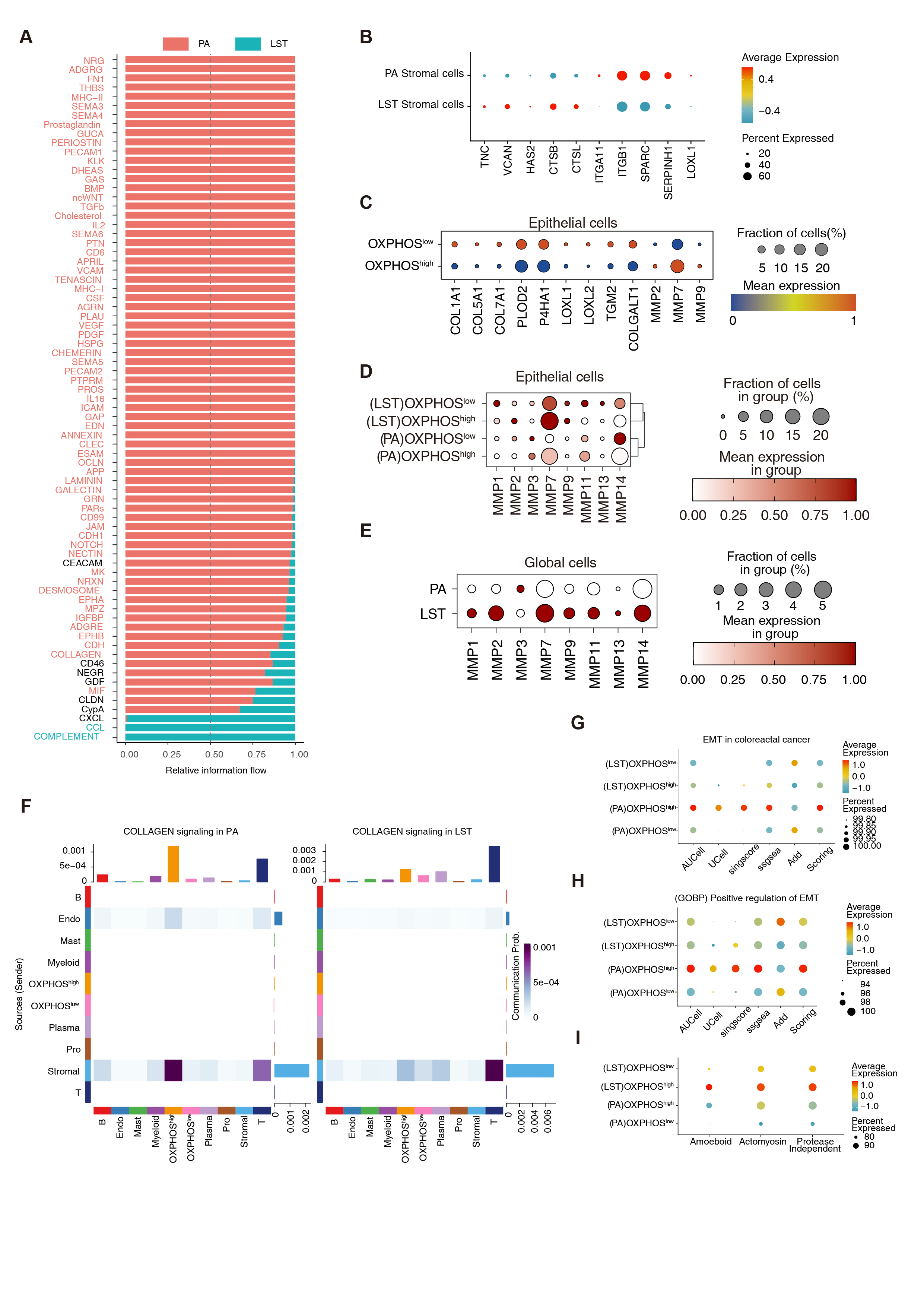

Supplement: Supplementary file 2 — Supporting File 2: advs74825‐sup‐0002‐FigureS1‐S8.zip. [file ADVS-13-e23872-s002.zip › sFig-7.jpg]

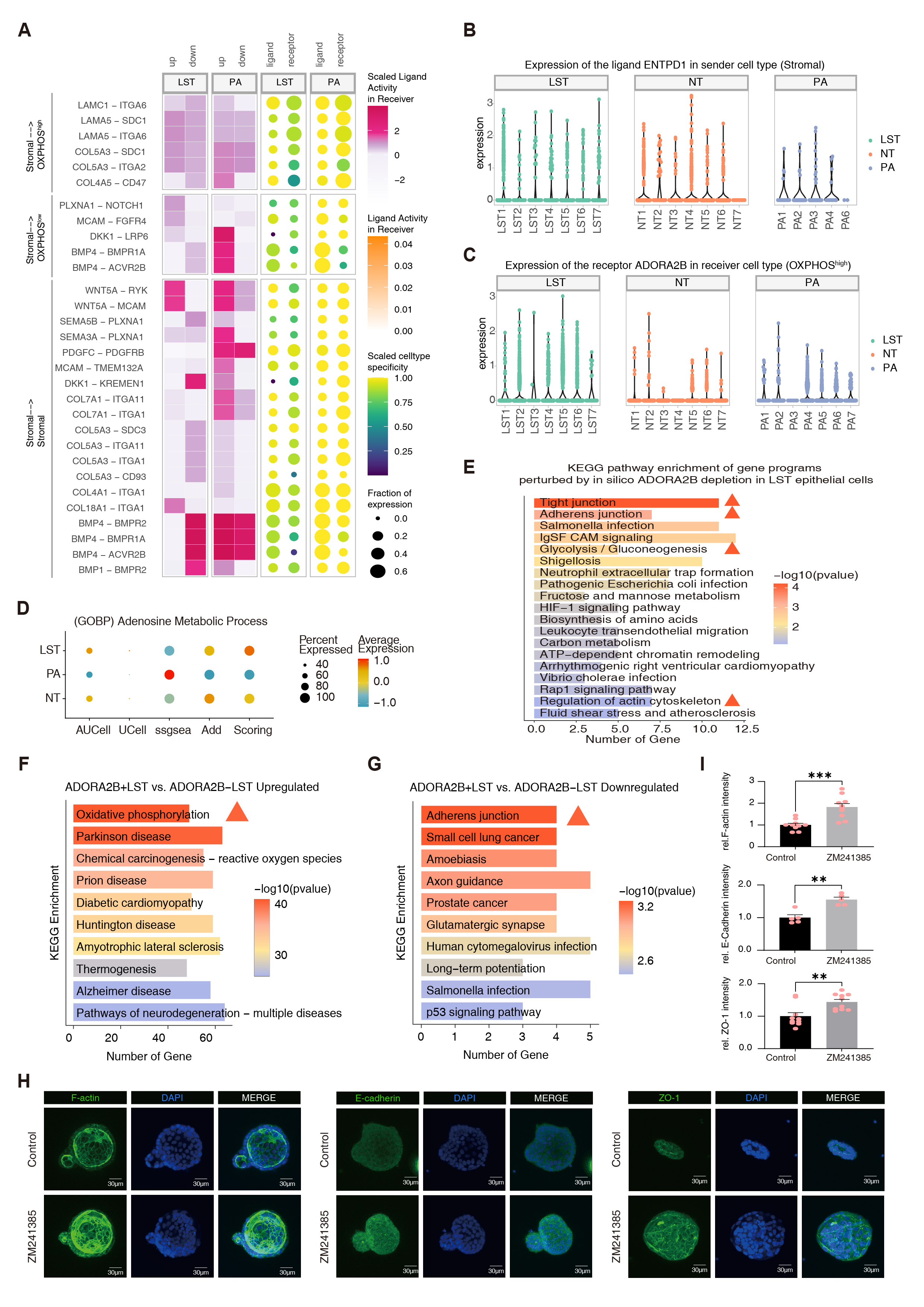

Supplement: Supplementary file 2 — Supporting File 2: advs74825‐sup‐0002‐FigureS1‐S8.zip. [file ADVS-13-e23872-s002.zip › sFig-8.jpg]
