# Supplementary material for: A Soft Matrix Microenvironment Promotes Laterally Spreading Tumors via Oxidative Phosphorylation‐Dependent Cell Adhesion
Source: Adv Sci (Weinh). 2026 Mar 15;13(30):e23872. doi: 10.1002/advs.202523872 (PMC13248847; doi:10.1002/advs.202523872)
Supplement: Supplementary file 3 — Supporting File 3: advs74825‐sup‐0003‐TableS1‐S5.zip. [file ADVS-13-e23872-s003.zip › Table S5.docx]

Univariate and multivariate analysis of factors affecting submucosal invasion

| **Characteristics** | **Univariate analysis** | | **Multivariate analysis** | |
| --- | --- | --- | --- | --- |
|  | **OR (95%CI)** | **p value** | **OR (95%CI)** | **p value** |
| Group |  |  |  |  |
| PA group | Reference |  | Reference |  |
| LST group | 36.91 (8.87-153.49) | <0.001 | 23.39 (5.10-107.26) | <0.001 |
| Age at treatment |  |  |  |  |
| <45 years | Reference |  | Reference |  |
| ≥45 years | 2.32 (0.71-7.57) | 0.162 | 1.45 (0.44-4.80) | 0.541 |
| Gender |  |  |  |  |
| Male | Reference |  | Reference |  |
| Female | 1.35 (0.71-2.56) | 0.361 | 1.06 (0.55-2.03) | 0.870 |
| Location of tumors |  |  |  |  |
| Right colon | Reference |  | Reference |  |
| Left colon | 2.90 (1.34-6.25) | 0.007 | 2.36 (1.08-5.13) | 0.031 |
| Unknown | 3.32 (1.10-9.95) | 0.033 | 4.78 (1.56-14.66) | 0.006 |
| Lesion size |  |  |  |  |
| <20 mm | Reference |  | Reference |  |
| ≥20 mm | 9.78 (4.83-19.80) | <0.001 | 2.07 (0.97-4.43) | 0.060 |
